# Cyclic nucleotide binding proteins in the *Arabidopsis thaliana *and *Oryza sativa *genomes

**DOI:** 10.1186/1471-2105-6-6

**Published:** 2005-01-11

**Authors:** Dave Bridges, Marie E Fraser, Greg BG Moorhead

**Affiliations:** 1 Department of Biological Sciences, University of Calgary, 2500 University Dr. N.W. Calgary, AB, T2N 1N4 Canada

## Abstract

**Background:**

Cyclic nucleotides are ubiquitous intracellular messengers. Until recently, the roles of cyclic nucleotides in plant cells have proven difficult to uncover. With an understanding of the protein domains which can bind cyclic nucleotides (CNB and GAF domains) we scanned the completed genomes of the higher plants *Arabidopsis thaliana *(mustard weed) and *Oryza sativa *(rice) for the effectors of these signalling molecules.

**Results:**

Our analysis found that several ion channels and a class of thioesterases constitute the possible cyclic nucleotide binding proteins in plants. Contrary to some reports, we found no biochemical or bioinformatic evidence for a plant cyclic nucleotide regulated protein kinase, suggesting that cyclic nucleotide functions in plants have evolved differently than in mammals.

**Conclusion:**

This paper provides a molecular framework for the discussion of cyclic nucleotide function in plants, and resolves a longstanding debate about the presence of a cyclic nucleotide dependent kinase in plants.

## Background

The discovery of cyclic 3'5'-adenosine monophosphate (cAMP) by Earl Sutherland in the late 1950s was one of the most significant paradigm shifts in biochemistry [1]. This breakthrough ushered in the concept of second messengers: intracellular molecules which transmit signals in cells and are derived from an extracellular signal. In the past half century, cyclic nucleotides (both cAMP and cGMP) have been implicated in a vast array of biological phenomena in all kingdoms of life [2].

The ubiquitous presence of cyclic nucleotides may be due to several characteristics which make it an ideal second messenger. Cyclic nucleotides are derived in a energetically favourable reaction from common metabolites (ATP and GTP), and can be broken down into non-toxic products (inorganic phosphate and AMP/GMP). The synthesis and degradation of cyclic nucleotides are controlled by enzymes termed adenylate (or guanylate) cyclases and cyclic nucleotide phosphodiesterases, respectively [3,4].

In plants, cyclic nucleotides have endured a checkered research history fraught with complications and setbacks. Despite this, recent work has shown unequivocally that cyclic nucleotides are present in plant cells [5,6], and that they play key roles in the regulation of plant physiology [7-9]. Furthermore, the recent identification and cloning of adenylate and guanylate cyclases in plants [7,10] may eventually give clues as to what signals the synthesis and degradation of these molecules in plants.

Cyclic nucleotides are able to bind to two distinct protein domains, CNB domains and GAF domains. CNB domains were first identified in the regulatory subunit of mammalian cAMP-dependent protein kinase (RI and RII). Since several CNB domain containing plant proteins have been shown to be directly modulated by cyclic nucleotides, this indicates that the CNB domain in plants is functionally similar to CNB domains in other organisms [11-15].

GAF domains were initially identified as conserved domains in light sensing molecules but are known as small molecule binding domains in cyclic nucleotide regulated phosphodiesterases, the *Anabaena *cyclic nucleotide stimulated adenylate cyclase and several other proteins [16]. GAF domains have been shown to bind both cAMP [17] and cGMP [18,19]. Recent crystal structures of the GAF domains of human PDE2 [pdb:1MC0] and the yeast protein YKG9 [pdb:1F5M] have shown that this domain is an alpha/beta two layer sandwich with no structural or sequence homology to the CNB domain [18,20]. Therefore, GAF and CNB domains have evolved independently to bind cyclic nucleotides.

In order to further explore the roles of cyclic nucleotides in plants, we performed a bioinformatics based analysis of the completed *Arabidopsis thaliana *and *Orzya sativa *genomes [21-23] in order to elucidate the potential targets of cyclic nucleotides in plants.

## Results and discussion

### GAF domains

Based on the PDE2 crystal structure 11 residues were proposed to be involved in cyclic nucleotide binding [18], but comparison to the cAMP binding GAF domain of the *Anabaena *adenylate cyclase shows that these residues may only be strictly conserved in mammals. Further complicating our analysis is the fact that GAF domains are known to bind other small molecules such as 2-oxoglutarate, formate and bilins [24-26]. GAF domains form a structural scaffold which can be utilized to bind several possible small molecules depending on the functional groups on that scaffold. Therefore, their role in cyclic nucleotide signalling must be verified by biochemical means rather than strictly by sequence analysis. Our analysis indicated that in plants there are two types of proteins which contain GAF domains. These are the phytochrome proteins and the ethylene receptor proteins.

### Phytochromes

Phytochromes are light sensing signal transduction molecules which function to control several aspects of plant biology. Interestingly phytochromes were found to function upstream of cyclic nucleotides in their signal transduction pathways since their functions can be mimicked by cGMP and calcium in phytochrome knockout cells [27-29]. The light sensing portion of the phytochrome molecule is a covalently linked bilin molecule which is known to be bound by the GAF domain. Therefore it is unlikely that the GAF domain of the phytochrome is also able to bind cyclic nucleotides directly, although it is clear that cyclic nucleotides are somehow involved in this signalling pathway.

### Ethylene receptors

Ethylene responses have been documented for nearly a century in plants. This gaseous hormone is involved in many aspects of plant physiology, including fruit ripening, organ development, germination, seedling growth, flowering and response to challenges such as pathogens and stress [30]. There are five putative ethylene receptor isoforms in both Arabidopsis and rice as determined by genome sequencing [31]. All known ethylene receptors contain a GAF domain in a cytoplasmic region amino-terminal to the kinase domain. It has been speculated that this domain may be involved in cyclic nucleotide signalling but examination of heterologously expressed, functional ETR1 [Swiss-Prot: P49333] showed no detectable cyclic nucleotide binding (G. E. Schaller, personal communication). There are other ethylene receptors which have GAF domains and which to our knowledge have not been tested for cNMP binding, however, to date there is no evidence of cNMP regulation of ethylene receptors. Currently the function and ligands of the GAF domain in ethylene receptors is unknown.

### CNB domains

**Figure 1:**
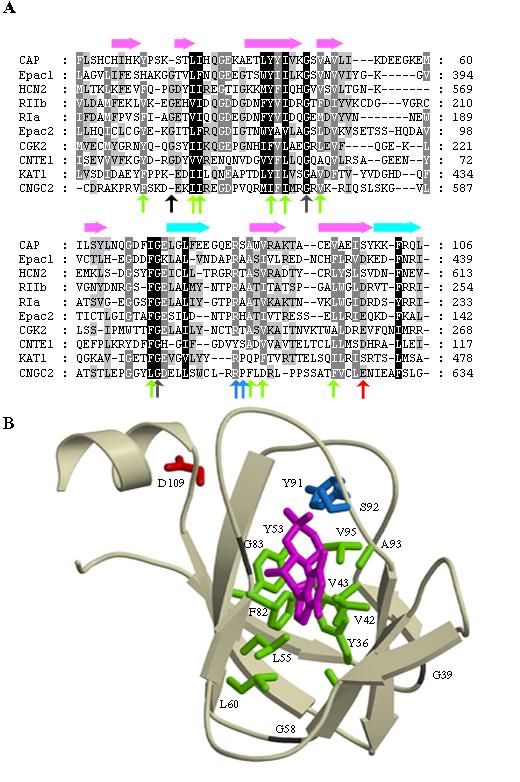
**Analysis of plant CNB domains**. (A) Arabidopsis CNB domains (CNTE1, KAT1 and CNGC2) were aligned against several well studied CNB domains including regulatory subunits of PKA (RIα and RIIβ), Epac1, Epac2, and cyclic GMP dependent kinase 2 (CGK2) from humans, HCN2 from mouse and *E. coli *CAP. Highlighted on the alignment are glycine residues involved in loop structures (dark grey arrows), residues forming the hydrophobic pocket for cNMP binding (green arrows) and residues proposed to contact the phosphate of the cNMP (blue arrows). The highly conserved helix capping acidic residue is shown in red. Secondary structure is denoted by arrows above the alignment, with light blue for alpha helices and pink for beta sheets and is based on the secondary structure of HCN2. (B) A homology model of atCNTE1 was generated from the known structures of CNB domains. Key residues are shown as stick representations and are colored and labeled according to the color scheme described in (A). The cGMP ligand is shown in magenta and is based on the structure of cGMP bound to HCN2 [pdb: 1Q3E] superimposed over our model. Figure was generated with Molscript [83] and Raster3D [84].

From the alignment of CNB domains in animals, bacteria and plants, it was apparent that there are some strong similarities, as well as some significant differences (Figure 1A). In order to visualize whether plant CNB domains could fold in a similar manner to the other well characterized CNB domains, we generated an *in silico *model. We chose the plant protein which showed highest similarity to known crystal structures (*Arabidopsis thaliana *CNTE1) and based our model on the solved crystal structures of RIα, RIIβ, HCN2, CAP and Epac2 (Figure 1B and see additional file 5).

We then examined our model's overall topology as well as its cyclic nucleotide binding site. The basic fold of the domain is two anti-parallel beta sheets consisting of four strands forming a sandwich, ending with an alpha helix (the hinge region). Connecting these sheets are exposed loops, the most important of which is the phosphate binding cassette [32]. It is important to note that our structure models very well against all CNB domains with excellent conservation of all secondary structure and most loops. We calculated the backbone root mean square deviation for our model versus each of the templates as: RIIβ domain 1: 0.76 Å; RIIβ domain 2: 0.83 Å; Epac1 domain 1: 1.08 Å; RIα domain 1: 0.85 Å; RIα domain 2: 0.94 Å; HCN2: 0.82 Å and CAP: 1.12 Å. Our model agrees in general with a previously reported model for the Arabidopsis CNGC2 [33], although a detailed comparison between the two models was not performed. The use of a less distant target (atCNTE1) as well as several templates (seven compared to one) adds to the reliability of our structure. In all mammalian cAMP-binding structures solved, there is a key arginine residue (Arg 209 in RIα) which forms a salt bridge with the cNMP's phosphate group. This residue is absent in some CNB domains, despite evidence that at least some of these domains do in fact bind cyclic nucleotides. For example, this residue is absent in the Drosophila ether-a-go-go channel, which is known to be modulated by cyclic nucleotides [34]. Examination of our model shows that in the region near the phosphate, there are two residues which may functionally replace the arginine, Tyr 91 and Ser 92 (Figure 1B). In bacterial CAP, hydrogen bonding is provided to the phosphate by the Arg 82 sidechain, Ser 83 amide nitrogen atom and sidechain hydroxyl group, as well as a water molecule. In some mammalian isoforms, the serine residue is changed to an alanine and therefore is only able to provide backbone hydrogen-bonding. In our plant atCNTE1 model, the serine residue is conserved, but the arginine residue is missing. Since there was no good template to model the phosphate binding cassette onto, our model only approximates the position of this loop, and will require verification by other structural studies.

The hydroxyl and amide groups of Ser 92, as well as the hydroxyl group of Tyr 91 are all within proximity of the cNMP phosphate and could play a role in stabilizing the cyclic nucleotide (see blue residues in Figure 1). Examination of the region which contacts the base, indicates that our model is most similar to the structure of CAP in this region, so it is likely that the base moeity of a cNMP is bound in a syn orientation as in CAP. This is in agreement with a previously reported atCNGC2 model [33]. Further analysis of the binding site for the nucleotide indicates that it is likely cGMP which binds to atCNTE1. This conclusion is based upon the presence of three residues (Tyr 80, Ser 92 and Ser 109) which could potentially differentiate between cyclic nucleotides, each of which has a preference for cGMP (Figure 1 and [35,36]). Other conserved structural features of our model are the hydrophobic pocket forming residues Tyr 36, Val 42, Val 43, Tyr 53, Leu 55, Ala 60, Phe 82, Ala 93 and Val 95 and Leu 105 (see green residues in Figure 1) as well as several conserved glycine residues which are involved in turns between the beta strands (39, 58 and 83) and the helix capping Asp 109. This residue signals the end of the hinge region alpha helix and is present in most CNB domains examined.

**Figure 2:**
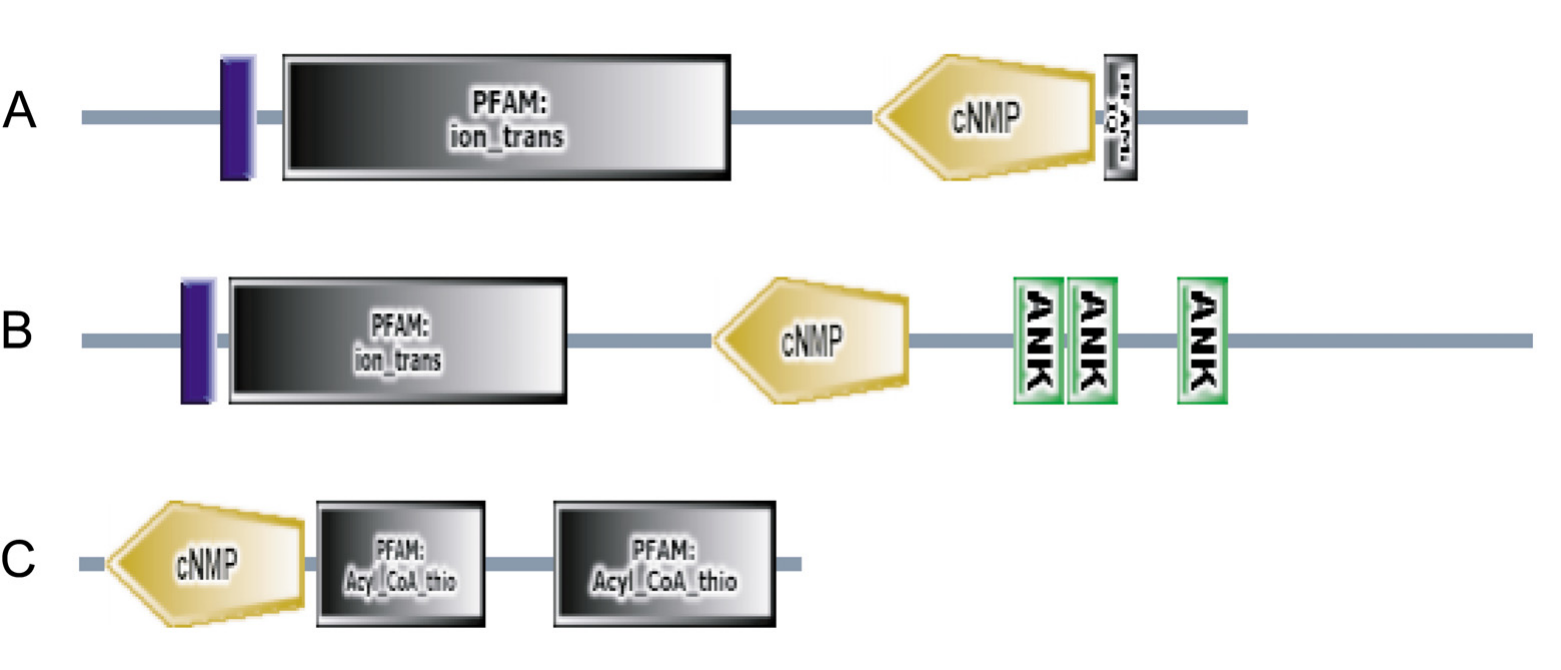
**Domain structures of representative CNB domain containing proteins in plants**. Scaled images were generated using SMART [60, 62] A) atCNGC2 showing the ion channel, CNB domain and IQ (Calmodulin binding) domain. B) AKT1 showing the ion channel, CNB domain and Ankyrin repeats (ANK). C) atCNTE1 showing the CNB domain and the Acyl-CoA thioesterase domain.

**Figure 3:**
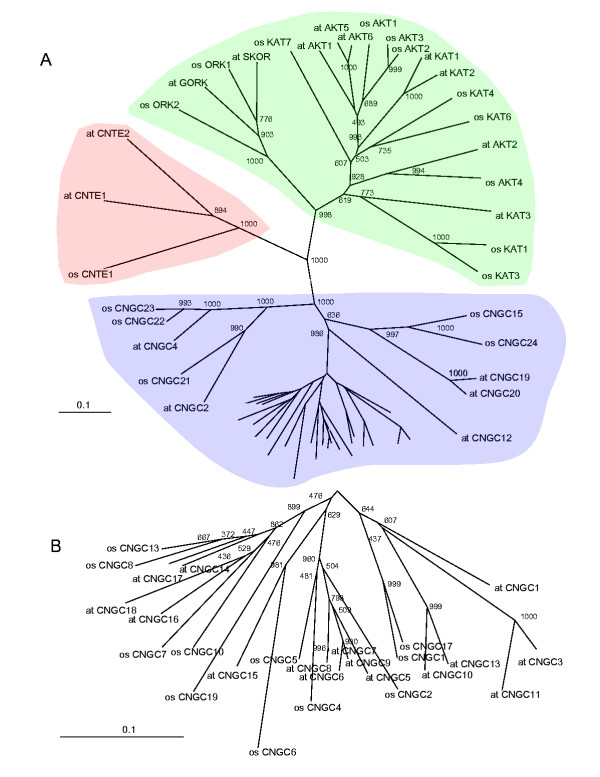
**Phylogenetic analysis of plant CNB domains**. Un-rooted neighbor-joining tree of CNB domains in *Arabidopsis thaliana *and *Oryza sativa*. Shading represents the three classes of CNB domains in plants; shaker-type potassium channels are shown in green, acyl-coA thioesterases are shown in red and CNGC channels are shown in blue. Numbers on the nodes indicate the number of possible trees out of 1000 in which that node was present. Scale represents the number of differences per residue. B) This is an expanded view of the unlabelled region in Figure 3A showing the closely related CNGC proteins more clearly. Tree was generated using ClustalX [65] and visualized using TreeView [68]. See additional file 4 for sequence alignments.

Phylogenetic analysis indicates that the plant CNB domains segregate into three subfamilies (Figures 2 and 3). The phylogenetic distribution of the CNB domain matches their domain context in that CNGC, shaker-type and CNTE proteins form separate groups. Furthermore, for each of the three protein classes, the CNB phylogeny matches the phylogeny of the full-length protein, implying that these proteins obtained the CNB domain prior to isoform duplication (Figure 3, [Additional file 1], [37]). Since all three branches have been detected in both Arabidopsis and Oryza, it is likely that the specific plant cyclic nucleotide responses developed prior to monocot-dicot divergence. We did not find CNB domains in any protein kinases, transcription factors or guanine nucleotide exchange factors in our analysis. Each of the three classes of CNB domain containing proteins will be discussed below.

### Cyclic nucleotide gated ion channels

Plant CNGC ion channels were first identified in a screen for calmodulin binding partners in barley [38]. There are now known to be 20 CNGC proteins in Arabidopsis likely indicating a high level of channel redundancy [37,39]. We also detected 16 CNGC proteins in rice by examination of the TIGR Rice Genome Annotaion Resource [40].

Electrophysiological studies have shown the CNGC channels to be permeable to potassium, sodium and calcium [13-15,41-43]. Cyclic nucleotides have been shown to activate channel opening in all CNGC proteins examined thus far leading to an influx of cations into the cell [13-15,33]. Mutagenic screens have shown that mutations in atCNGC2 and atCNGC4 create faulty pathogenic reactions [13,44]. When taken together with data showing that cyclic nucleotides are necessary for pathogen responses and that calcium and potassium influxes are characteristic of early phases of plant pathogen responses [45], this seems to imply that cyclic nucleotides may play a role in controlling plant immune responses.

Finally, work by Maathuis and Sanders [8] has shown that cyclic nucleotides can modulate sodium uptake in Arabidopsis plants, implying that there is a cyclic nucleotide controlled channel which plays a role in salinity tolerance. They showed that cyclic nucleotides are required for limiting sodium uptake in root protoplasts, but the exact molecule (cAMP or cGMP) responsible for this effect has not been pinpointed.

### Shaker-type potassium channels

Plant potassium channels fall into two classes, the KCO channels and the shaker-type channels [37]. In addition to the 9 shaker-type channels described in *Arabidopsis thaliana*, we have found 10 channels in *Oryza sativa*. A variety of mutational studies have implicated the shaker-type channels in several key processes involving the movement of potassium including: from the soil (AKT1, KAT3), long distance transport (AKT2), transport into growing pollen tube (AKT6), secretion into xylem sap (SKOR) and transport during guard cell opening either into the cell (KAT1, KAT2) or out of the cell (GORK) [37].

Shaker-type channels are voltage dependent outward (GORK, SKOR) or inward (KAT and AKT) rectifying channels. Analysis of heterologously expressed channels have shown that cyclic nucleotides function to adjust the activation potential of these channels [11,12]. Since cyclic nucleotides have already been implicated in some of the processes controlled by shaker-type channels [7-9,46], it is reasonable to believe that cyclic nucleotides are physiological regulators of shaker-type potassium channels.

### Cyclic nucleotide regulated thioesterases

Initially we detected a short CNB containing protein which was only slightly larger than the domain itself. Sequencing of the EST provided by the Arabidopsis Biological Resource Center [47] showed the protein was actually mis-annotated by the automated gene-finding algorithm. Further analysis indicated that there are two isoforms of this protein in Arabidopsis and one in rice. Each protein contains an amino-terminal CNB domain and a carboxy-terminal acyl-CoA thioesterase domain. Searches of other partially sequenced plant genomes and EST databases indicated that these proteins are present in several plant species, but not in any other division of life and thus represents a novel plant-specific cyclic nucleotide target. Comparison of these protein sequences indicates a high level of conservation, including residues conserved for both catalysis and cyclic nucleotide binding domain structure. Arabidopsis CNTE1 had previously been partially characterized as a thioesterase and shown to have activity versus both 16:0-CoA and 18:1-CoA when over-expressed and partially purified from *E. coli *[48].

Fatty acid synthesis requires the use of acyl-CoA's as building blocks for incorporation into lipids. It is therefore possible, that these thioesterases function as scavengers which remove "irregular" fatty acids from the pool of available building blocks [48]. Furthermore, the thioesterases could divert fatty acids away from biosynthetic pathways and β-oxidation during germination or during stressful conditions. In most cases when a small molecule binding domain is connected to a catalytic domain on the same polypeptide, the catalytic domain is regulated by the small molecule [49]. The conservation of this protein across planta indicates that the CNB domain likely has a role in controlling the thioesterase activity of this enzyme, but it is unknown at this time exactly what role cyclic nucleotides play in this process. In order to address this we cloned and tried to express the atCNTE1 protein in *E. coli*, however after extensive trials we were unable to express and purify soluble protein.

### A cyclic nucleotide dependent protein kinase in plants?

We found no PKG or PKA regulatory subunit homologs in the Arabidopsis genome. There has been a long standing controversy in the plant field as to the existence of a plant cyclic nucleotide dependent kinase [50-52]. As PKA is the major cAMP target in mammalian cells we chose a biochemical approach to further explore the possibility that a PKA-like enzyme may be present in Arabidopsis.

**Figure 4:**
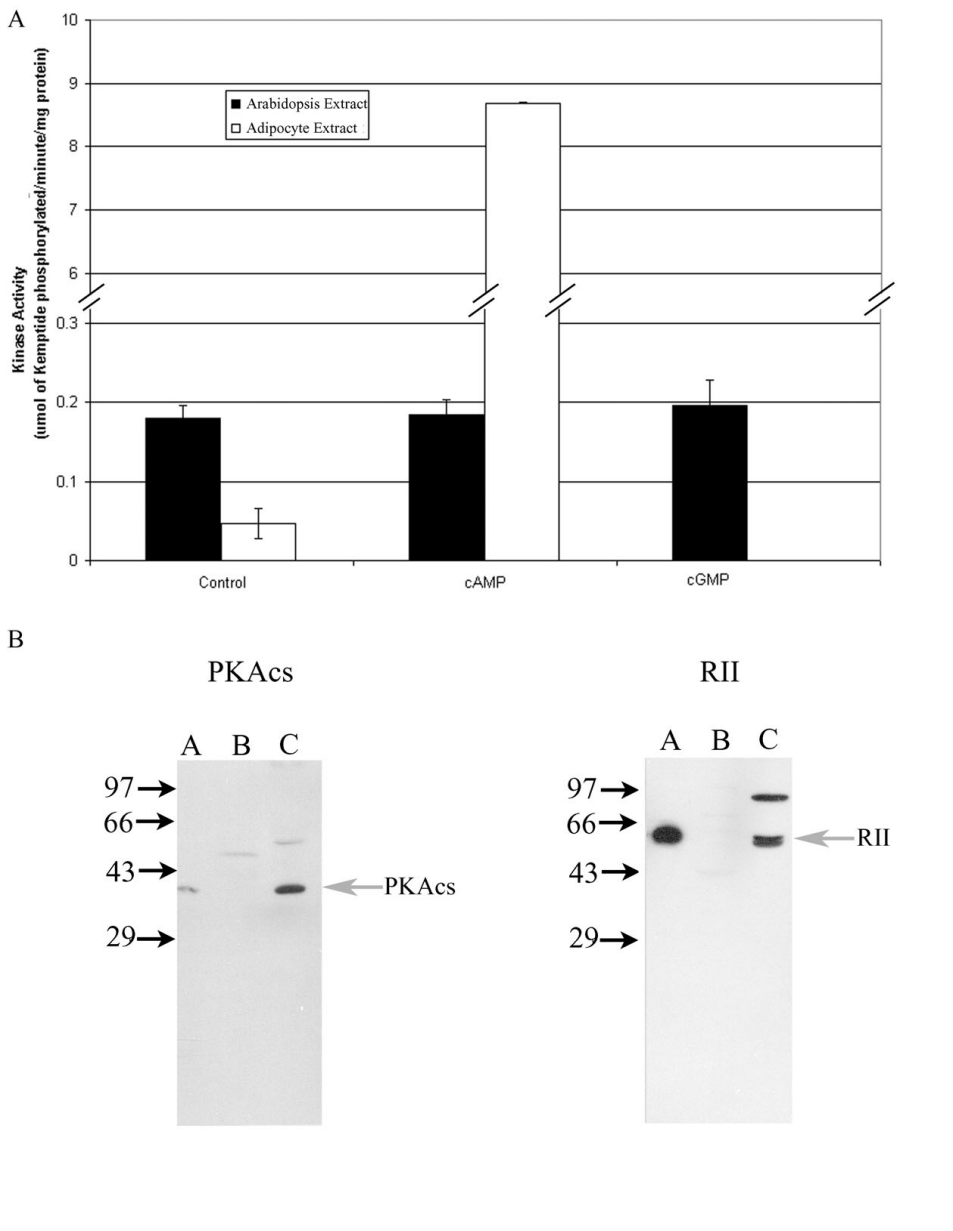
**Biochemical evidence for lack of a cyclic nucleotide dependent kinase in *Arabidopsis thaliana***. (A) Protein kinase assays using Kemptide as a substrate. Assays were conducted on identically prepared extracts of Arabidopsis and rat adipose tissue in the presence or absence (control) of 10 μM cyclic nucleotide as indicated. Scale is offset in order to visualize both sets of results. All assays were performed in duplicate from three separate preparations and error bars indicate standard error for three separate preparations. (B) Western blotting of extracts with PKA catalytic (PKAcs) and regulatory (RII) subunit polyclonal antibodies. The PKAcs antibody was affinity purified according to [82] and used at 0.5 μg/mL while the RII antibody was used as crude serum at 5000X dilution. Lanes are as follows (A), 10 ng of purified bovine PKAcs or RII, (B) 25 μg clarified crude Arabidopsis extract, (C) 25 μg clarified crude rat adipocyte extract. Positions of mammalian PKA and RII are indicated with arrows.

We performed protein kinase assays with extracts of *Arabidopsis thaliana *using the PKA substrate Kemptide. Kemptide is a peptide which has a motif which was confirmed as the optimal substrate for PKA [53,54] and is routinely used in mammalian PKA assays. As Figure 4A shows, there was no detectable increase in kinase activity in the plant cell extracts when cAMP or cGMP are added. Fractionation of extracts, as well as testing a range of cyclic nucleotide concentrations also did not allow us to detect any differences in kinase activity with addition of cyclic nucleotides (data not shown). For comparison, adipocyte extracts (a cAMP responsive mammalian tissue) were assayed as well, illustrating the large increase in protein kinase activity in these cells when cAMP is added. Furthermore, blotting of *A. thaliana *extracts with polyclonal antibodies raised against mammalian PKA subunits (both the catalytic and the RIIα subunit) reveals that no structurally similar proteins are present in this extract (Figure 4B). Blotting with a monoclonal antibody to the RIIβ subunit gave similar results (not shown). Although there is a weak band present in the Arabidopsis extract which cross-reacted with the catalytic subunit polyclonal antibody, it is likely unrelated to cyclic nucleotide signalling. Protein kinase catalytic domains are very highly conserved [55,56] and therefore a minor amount of cross-reactivity is not unexpected. Further adding to the validity of the western blotting experiment is the observation that several studies have shown that true PKA-like enzymes in non-mammalian eukaryotes do cross react with antibodies raised against the regulatory subunits of mammalian PKA [57,58], whereas this is not detected in our plant extracts.

The lack of evidence for kinase activity could be attributed to substrate specificity, differences in binding affinity or expression levels in plant extracts relative to mammalian extracts, so our experimental approach does not exhaustively rule out the possibility of a cNMP dependent kinase activity. However, we feel that these data in concert with the genomic and blotting data strongly suggest that there is no cNMP dependent kinase in plants. Finally, our data imply that if such a protein exists, it would bear little or no sequence, structural or biochemical similarity to the classically studied mammalian enzyme.

## Conclusions

As the understanding of cyclic nucleotide signalling in a variety of systems has progressed, it has been increasingly difficult to describe a general role for cyclic nucleotides in biology. They control 'well-fed' gene transcription in bacteria, and modulate signal transduction and ion currents in mammals, resulting in a large number of possible physiological responses.

This analysis is potentially limited in that it only analyses cNMP domains which have already been previously identified and characterized in other systems. However, conservation of CNB and GAF domains as the only known cyclic nucleotide binding domains present over a wide cross-section of life indicates that these domains are likely to control most, if not all cyclic nucleotide responses. It is possible however, that plants have evolved entirely novel domains which can be modulated by these second messengers. It will be interesting to compare this *in silico *analysis with future biochemical data regarding the direct effectors of cyclic nucleotide signalling in plants. It is interesting that no homologous proteins in the CNGC, shaker-type or type II acyl-CoA thioesterase families have been found which lack CNB domains. This implies that cyclic nucleotide binding is indispensable to their cellular role.

Although it would have been interesting if this analysis revealed more novel classes of plant cyclic nucleotide binding proteins, the fact that (with the exception of CNTE) all cyclic nucleotide binding proteins had been previously identified indicates that the previously attained biochemical data agrees with our bioinformatic evidence.

The identification of no transcription factors or protein signal transduction molecules with CNB domains implies that cyclic nucleotides may be unable to directly modify the proteome of plant cells. This is in stark contrast to bacterial, yeast and mammalian systems. The only common domain context of CNB domains in animals and plants is the CNGC channels, however, even these channels appear to have evolved independently [39,59]. Therefore it is clear that the roles of cyclic nucleotides in prokaryotic and eukaryotic, as well as plant and animal systems differ and that evolutionarily distant branches of life have evolved different mechanisms by which these molecules are utilized. It is worth pointing out that the ubiquitous presence of cyclic nucleotides in all forms of life may indicate that although the means by which this particular biochemical tool is used differ, it is still an indispensable component of biology's toolbox.

## Methods

### Bioinformatics

In order to identify the proteins which contain CNB or GAF domains, we initially used the Simple Modular Architecture Research Tool (SMART at smart.embl.heidelberg.de; [60-62]) to scan all predicted Arabidopsis proteins for CNB and GAF domains in the EMBL, TIGR or NCBI databases. Once redundancies were removed, a list of proteins was generated [see additional file 2]. In order to ensure broad coverage of possible variants, we also examined the Interpro collection of protein sequence analysis algorithms, all of which use slightly different methods [63]. As an additional method, the predicted proteins of the Arabidopsis genome were searched using the BLAST algorithm [64]. As search bait, we used several known cyclic nucleotide binding domains including those from GAF domains (human PDE2A [Swiss-Prot: O00408] and Anabaena cyaB1 [Trembl: P94181]) as well as CNB domains (human CGK2 [Swiss-Prot: Q13237], human RIIβ [Swiss-Prot: P31323], human Epac2 [Swiss-Prot: Q8WZA2], human rod CNGC [Swiss-Prot: P29973] and *E. coli *CAP [pir: E86000]). This yielded no new inclusions to our list of proteins, but did confirm each of our previous entries. For examination of the *Oryza sativa spp*. Japonica genome we performed BLAST searches using the aforementioned baits, as well as each of the Arabidopsis proteins. This search was performed using the Blast utility of the TIGR rice database [40]. The criterion for inclusion was that the CNB or GAF domain had to match the consensus motif with an E-value of less than 0.5 over the entire domain as determined by SMART. For newly identified proteins from the *Orzya sativa*, we named them so that they agreed best with the nomenclature of Maser *et al*. [37] [see additional files 1, 2, 3]. Sequence alignments were performed using the ClustalX [65] or T-COFFEE algorithms [66]and then inspected visually. Neighbor-joining trees were generated by ClustalX or PHYLIP [67], then were visualized with TreeView [68]. Trees generated using a variety of analysis methods (parsimony, distance and maximum likelihood) yielded similar results to the neighbor-joining trees.

### Sequencing of atCNTE1

One protein, which appeared to contain only a cyclic nucleotide binding domain and no other motifs was found in the Arabidopsis database. We obtained the clone corresponding to this putative gene from the Arabidopsis Biological Resource Center and sequenced it. Sequencing was performed at the University of Calgary Core Sequencing Facility. We determined that the gene prediction algorithm which scanned the genome improperly predicted the intron/exon structure of this gene. The new gene, which we named cyclic nucleotide regulated thioesterase 1 (atCNTE1) was deposited in the NCBI database [GenBank: AY874170]. A subsequent BLAST search using this gene found another isoform of this gene in Arabidopsis (atCNTE2) and one isoform in Rice (osCNTE1) which we also included in our analysis.

### Theoretical model of atCNTE1

We generated a model of the atCNTE1 cyclic nucleotide binding domain (residues 28–117) based on an alignment of atCNTE1 with the CNB domains of RIIβ [pdb: 1CX4] [69], RIα [pdb: 1RGS] [70], CAP [pdb: 1CGP] [71], HCN2 [pdb: 1Q3E] [72] and Epac1 [pdb: 1O7F] [32]. This was submitted to the SWISS-MODEL server via the DeepView program [73]. The alignment was iteratively refined to allow for best agreement of sequence and structural similarity.

### Cyclic nucleotide dependent protein kinase assays

Unless otherwise indicated all chemicals were purchased from Sigma-Aldrich. Assays were performed on extracts of Arabidopsis cells grown in suspension culture [74] or isolated male Wistar rat adipocytes from epididymal fat pads [75]. Both cell types were homogenized in 50 mM Tris pH 7.5, 5% (v/v) glycerol, 0.2 mM phenylmethylsulfonyl fluoride, 1 mM benzamidine and 0.1% (v/v) 2-mercaptoethanol. Adipocytes were lysed by 10 strokes of a dounce homogenizer while plant cells were lysed by two passages through a french press cell at 15 000 psi. The extracts were clarified by centrifugation for 15 min at 4000 RPM in a SS34 rotor at 4°C. These extracts were assayed for kinase activity using ^32^P-γ-ATP (Amersham-Pharmacia), 30 μM Kemptide substrate, 50 mM HEPES pH 7.4, 1 mM dithiothreitol and 10 μM cyclic nucleotide as specified. Reactions were allowed to occur for 10 minutes at 30°C and assays were terminated by spotting onto squares of P81 paper followed by extensive washing with 75 mM phosphoric acid [76]. Assays were performed in duplicate on three separate preparations with error bars indicating the standard error between preparations (n = 3). Protein concentration was determined by the method of Bradford with bovine serum albumin (ICN Biomedicals) as a standard [77].

### Western blotting of extracts

Bovine heart PKA catalytic subunit and RII were purified to homogeneity [78,79]. Purified protein was injected into rabbits and serum was obtained according to standard methods [80]. Extracts of adipose and plant cells were prepared as described above. Samples were boiled into SDS-PAGE buffer and separated on a 10% denaturing gel [81]. The proteins were then transferred to nitrocellulose for 2 h at 100V and blocked overnight with 5% (w/v) skim milk powder. Blots were probed with antibodies for 1 h and visualized by enhanced chemiluminesence. The PKA catalytic subunit antibody was affinity purified according to [82] and used at 0.5 μg/mL while RII was used as crude immune serum at a 5000X dilution. For the RII western blots, both a polyclonal and a monoclonal antibody (anti-RIIβ BD Transduction Laboratories) gave identical results.

## List of abbreviations

CAP catabolite activator protein

cAMP 3'5'-cyclic adenosine monophosphate

CGK2 cGMP dependent protein kinase 2

cGMP 3'5'-cyclic guanosine monophosphate

cNMP 3'5'-cyclic nucleotide (cAMP or cGMP)

CNB cyclic nucleotide binding

CNGC cyclic nucleotide gated channel

CNTE cyclic nucleotide dependent thioesterase

Epac exchange protein directly activated by cAMP

GAF domain found in cGMP-phosphodiesterases, adenylyl cyclases and FhlA

GORK guard cell outward rectifying potassium channel

PKA protein kinase A

SKOR stellar potassium outward rectifying channel

SMART simple modular architecture research tool

## Authors' contributions

DB performed the bioinformatic analysis, modeling and biochemical studies and drafted the manuscript. MEF participated in the modeling. GBG conceived of the study and participated in its design and co-ordination. All authors have read and approved the final manuscript.

## Supplementary Material

Additional File 5 **Co-ordinate file of model generated for Figure **1b. Click here for file

Additional File 1 **Phylogenetic analysis of CNGC and shaker-type channels from *Arabidopsis thaliana *and *Oryza sativa*. **Un-rooted neighbor-joining trees were constructed for (A) shaker-type and (B) CNGC channels using full-length sequences. For the shaker-type channels, numbers at nodes indicate number of trees out of 100 in which the node occurred. These are omitted in (B) for clarity. Scale indicates number of differences per residue. Trees were generated using ClustalX [65] and visualized with TreeView [68]. Click here for file

Additional File 4 **Relevant Sequence Alignments **Protein sequence alignments upon which phylogenetic analyses in Figure 2, and additional file 2 are based. Click here for file

Additional File 2 **Cyclic nucleotide binding proteins in *Arabidopsis thaliana *and *Oryza sativa*. **Proteins containing CNB domains including species, name, aliases, accession number, other domains present (AR indicates ankyrin repeat, CaM indicates calmodulin binding domain and TE indicates type II acyl CoA thioesterase domain), sequence length, residues encompassing the CNB domain and E-value for the CNB domain as determined by SMART [62]. All accession numbers are from NCBI except osCNGC5b, osCNGC5c and osCNGC18 which were obtained from the MIPS Oryza sativa database . Click here for file

Additional File 3 **Protein sequences of all sequences analysed in this manuscript. **Sequences are named according to details in the text and are in FASTA format. Click here for file
