# Supplementary figures and images for: Cyclic nucleotide binding proteins in the Arabidopsis thaliana and Oryza sativa genomes
Source: BMC Bioinformatics. 2005 Jan 11;6:6. doi: 10.1186/1471-2105-6-6 (PMC545951; doi:10.1186/1471-2105-6-6)

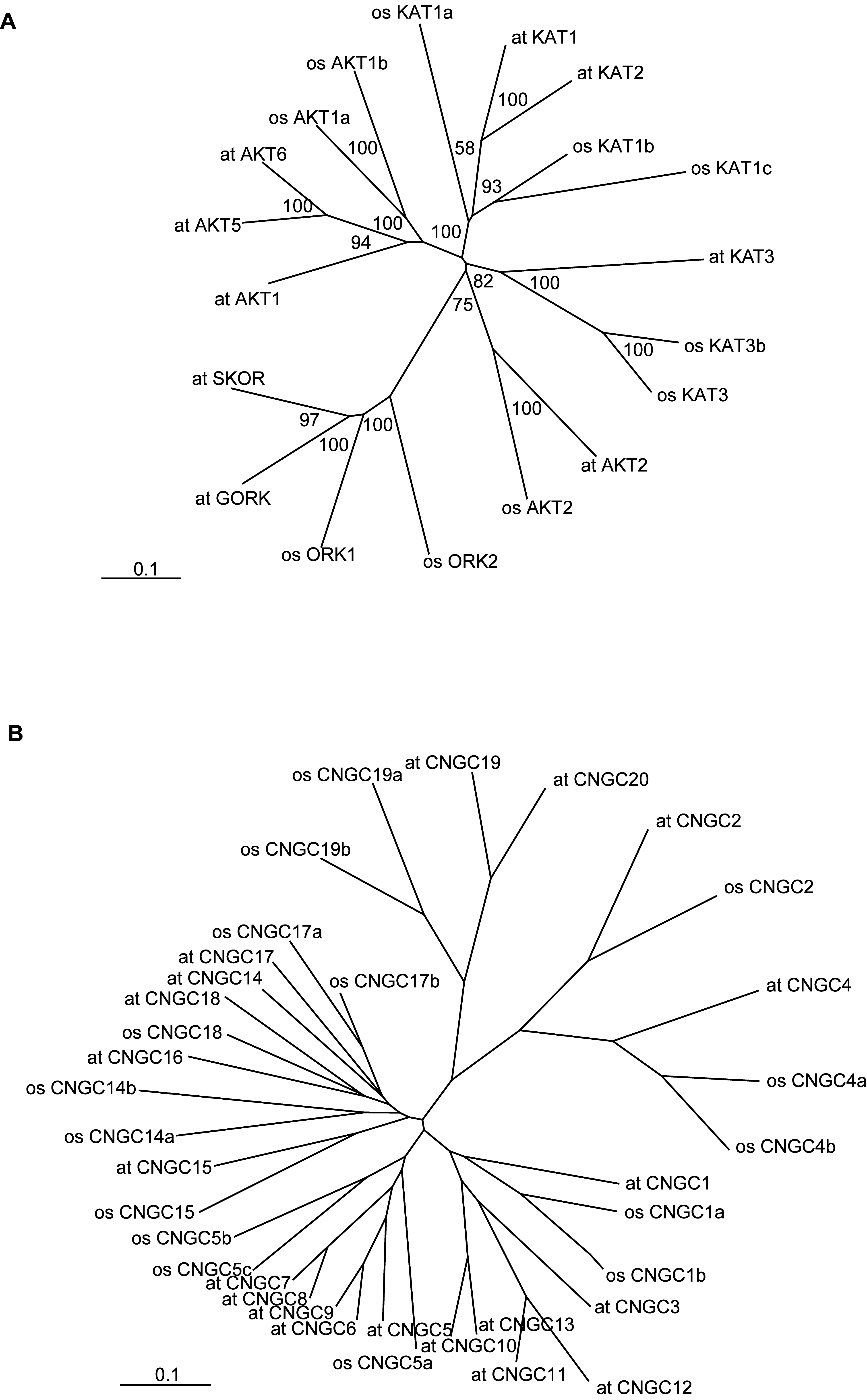

Supplement: Additional File 1 — Phylogenetic analysis of CNGC and shaker-type channels from Arabidopsis thaliana and Oryza sativa. Un-rooted neighbor-joining trees were constructed for (A) shaker-type and (B) CNGC channels using full-length sequences. For the shaker-type channels, numbers at nodes indicate number of trees out of 100 in which the node occurred. These are omitted in (B) for clarity. Scale indicates number of differences per residue. Trees were generated using ClustalX [65] and visualized with TreeView [68]. [file 1471-2105-6-6-S1.tiff]
