# Supplementary material for: Cyclic nucleotide binding proteins in the Arabidopsis thaliana and Oryza sativa genomes
Source: BMC Bioinformatics. 2005 Jan 11;6:6. doi: 10.1186/1471-2105-6-6 (PMC545951; doi:10.1186/1471-2105-6-6)
Supplement: Additional File 4 — Relevant Sequence Alignments Protein sequence alignments upon which phylogenetic analyses in Figure 2, and additional file 2 are based. [file 1471-2105-6-6-S4.rtf]

Sequence Alignment for Figure 2 
                                                                                               
                      *        20         *        40         *        60         *              
at_CNGC16  : -FFAQMDD--------QLLDAICERLVPSLNTKDTYVIREGDPVNEMLFIIRGQMESSTTDGGRSGFFNSITLRPGDF :  69
at_CNGC18  : -FFSQMDD--------QLLDAICGCLVSSLSTAGTYIFREGDPVNEMLFVIRGQIESSTTNGGRSGFFNSTTLRPGDF :  69
os_CNGC18  : -FFSQMDE--------QLLDAICERLVSSLSTKDAYIVREGDPVSEMLFVIRGELESSTTDGGRTNFFSSITLRPGDF :  69
os_CNGC16a : -FFSEMDY--------QLLDAICERLVSFLCPERTYISREGDPVNEMLFVIRGKLESSTTNGGRSNFFNSIILRPGDF :  69
os_CNGC17a : -NVSLMDG--------QLLDAICERLVSSLSTVGTYIVREGDPVTEMLFIIRGKLESSTTDGGRTGFFNSITLKTGDF :  69
os_CNGC17b : -FFSQMDN--------QLLDAICERLVSSLCTQGTYIVREGDPVTEMLFIIRGKLESSTTNGGRTGFFNSTTLKSGDF :  69
at_CNGC14  : -LFAQMDD--------QLLDAICERLASSLSTQGNYIVREGDPVTEMLFIIRGKLESSTTNGGRTGFFNSITLRPGDF :  69
at_CNGC17  : -FFSQMDD--------QLLDAICERLVSSLCTEGTYLVREGDLISEMLFIIRGRLESSTTNGGRTGFFNSIILRPGDF :  69
os_CNGC16b : -FFSAMDH--------QLLDAICERMTYFLRTEGTYITREGDPVKVMLFIIRGKLESSTTDGGRTGFFNSIILKPGDF :  69
at_CNGC9   : -LFENMDE--------RLLDAICERLKPCLYTESSYLVREGDPVNEMLFIIRGRLESVTTDGGRSGFFNRSLLKEGDF :  69
at_CNGC6   : -LFENMDE--------RLLDAICERLKPCLFTEKSYLVREGDPVNEMLFIIRGRLESVTTDGGRSGFYNRSLLKEGDF :  69
at_CNGC5   : -LFKSMDD--------KLLDAICMRLKPCLFTESTYLVREGDPVDEMLFIIRGRLESVTTDGGRSGFFNRSLLKEGEF :  69
at_CNGC8   : -LFANMDE--------RLLDAICERLKPSLYTESTYIVREGDPVNEMLFIIRGRLESVTTDGGRSGFFNRGLLKEGDF :  69
at_CNGC7   : -LFANMDE--------RLLDAICERLKPSLFTESTYIVREGDPVNEMMFIIRGRLESVTTDGGRSGFFNRGLLKEGDF :  69
os_CNGC13  : -LFENMDE--------RLLDAICERLRPTLYTENEYILREGDPVDEMHFILHGCLESETTDGGRSGFFNKVQLKEGAF :  69
os_CNGC5c  : -LFANMDE--------RLLDAICERLRPALYTERTFIIREGDPVDQMLFIIRGCLESITTDGGRSGFFNRSLLEESDF :  69
os_CNGC5b  : -LFANMDE--------RLLDAICERLKPSLCTEATYILREGDPVDEMLFIIRGRLESSTTDGGRMGFFNRGLLKEGDF :  69
os_CNGC15  : -LFDEMDE--------RMLEAICERLRPALYTRGTRLVRELDPVDSMLFIIRGYLDSYTTQGGRSGFFNSCRIGAGEF :  69
at_CNGC15  : -LFDQMDE--------RMLDAICERLKPALCTEGTFLVREGDPVNEMLFIIRGHLDSYTTNGGRTGFFNSCLIGPGDF :  69
at_CNGC3   : -WFQAMDD--------RLLDALCARLKTVLYTEKSYIVREGEPVEDMLFIMRGNLISTTTYGGRTGFFNSVDLVAGDF :  69
at_CNGC11  : -LLQAMDD--------QLLDALCARLKTVHYTEKSYIVREGEPVEDMLFIMRGNLISTTTYGGRTGFFNSVDLIAGDS :  69
at_CNGC13  : -LFEIMDE--------QLLDAVCDKLKPVLYTENSYAIREGDPVEEMLFVMRGKLMSATTNGGRTGFFNAVYLKPSDF :  69
at_CNGC10  : -LFEIMDE--------QLLDAVCDRLRPVLYTENSYVIREGDPVGEMLFVMRGRLVSATTNGGRSGFFNAVNLKASDF :  69
at_CNGC1   : -MFEKMDE--------QLLDALCDRLQPVLYTEESYIVREGDPVDEMLFIMRGKLLTITTNGGRTGFLNSEYLGAGDF :  69
os_CNGC1a  : -MFENMDE--------KLLDAMCDRLKPMLYTEGSCIIREGDPVNEMLFIMRGNLESMTTNGGQTGFFNSNIIKGGDF :  69
os_CNGC1b  : -MFENMDD--------QLLNALCDRLKPVLYTEGSCIIREEDPVNEMLFIMRGNLMSMTTNGGRTGFFNSDVLKGGDF :  69
at_CNGC12  : ------DG--------WLLEAVCDRVKSVFYLANSFIVREGHPVEEMLIVTRGKLKSTTGSHEMGVRNNCCDLQDGDI :  64
at_CNGC2   : -LFRGMDD--------LILDNICDRAKPRVFSKDEKIIREGDPVQRMIFIMRGRVKRIQSLS--KGVLATSTLEPGGY :  67
os_CNGC2   : -LFHGMDD--------LILDNICDRLRPLVFSSGEKVIREGDPVQRMVFVLQGKLRSTQPLA--KGVVATCMLGAGNF :  67
os_CNGC4a  : -LFQHMDD--------LVLENICDRVKSLVFPKGEIIVREGDPVQRMLFIVRGHLQSSQVLR--TGATSCCTLGPGNF :  67
os_CNGC4b  : -LFHHMDD--------LVLENICDRVKSLISPKGEIIVREGDPVQRMLFIVRGHLQCSQVMR--NGATSWCTLGPGNF :  67
at_CNGC4   : -LFQHMDD--------LVLENICDRVKSLIFTKGETIQKEGDAVQRMLFVVRGHLQSSQLLR--DGVKSCCMLGPGNF :  67
os_CNGC19b : -LFNLMDN--------ATWDAICDKLRQNLYITGSDILYQGGPVEKMVFIVRGRLESISADG------NKSPLQEGDV :  63
os_CNGC19a : -LFTLMDW--------PILDAICDKLRQNLYISGSDILYQGGPVEKMVFIVRGKLESISADG------SKAPLHEGDV :  63
at_CNGC19  : -IFSLMDE--------SVLDSIRERLKQRTYIRSSTVLHHRGLVEKMVFIVRGEMESIGEDG------SVLPLSEGDV :  63
at_CNGC20  : -IFSLMDE--------PILDAIRERLKQRTYIGSSTVLHRGGLVEKMVFIVRGEMESIGEDG------SVLPLYEGDV :  63
at_CNTE1   : -LLQRLPS--------SSLKRISEVVVFKGYDRGDYVVRENQNVDGVYFLLQGQAQVLRSA--GEENYQEFPLKRYDF :  67
at_CNTE2   : -LLQKLPS--------SSLKKIAQVVVPKRYGKGDYVVREDQTWDGCYFILQGEAQVSGPD--EEDNRSEFLLKQYDY :  67
os_CNTE1   : -LLQCLPS--------SSIRRIADAVLVKRYEPGGYVAREGDPVDGLYIILDGQAEVSAPANTEEENRPDYVLNKYDY :  69
os_ORK1    : -LFKGCSA--------EFIQQIVIRLQEEFFLPGEVILEQGSAVDQLYFVCHGALEGVGIGEDGQEETILMLEPESSF :  69
at_SKOR    : -LFRGCSS--------EFINQIVIRLHEEFFLPGEVIMEQGSVVDQLYFVCHGVLEEIGITKDGSEEIVAVLQPDHSF :  69
at_GORK    : -LFKGCST--------EFINQIVIRLHEEYFLPGEVITEQGNVVDHLYFVCEGLLEALVTKTDGSEESVTLLGPHTSF :  69
os_ORK2    : -LFRGCSD--------DFLSQIVLKLHEEFFLPGEVILEQGTVVDQIYIVAHGCLEEVANGEDGSEEIISELRPYGIV :  69
at_AKT5    : -LFHGISN--------DLLFQLVSEMKAEYFPPKEDVILRNEAPSDFYIMVTGAVDIIARVNGVDQ-VVGEAQTGHVF :  68
at_AKT6    : -LFHGISN--------DLLFQLVTEMKAEYFPPKEDVILQNEAPTDFYILVTGAVDIIARVNGVEQ-VVSEAQRGHVF :  68
at_AKT1    : -LFRGVSN--------DLLFQLVSEMKAEYFPPKEDVILQNEAPTDFYILVNGTADLVDVDTGTES-IVREVKAGDII :  68
os_AKT1a   : -LLRTVTEYAYLVLLLDFVFE-VSEMKAEYFPPREDVILQNEAPTDFYILVSGSV------------VIQVATSGEVV :  64
os_AKT1b   : -LFEGVSN--------DLIFQLVSEMNAEYFAPREDIILQNEAPADFYIIVSGSM------------LAGMAKSGDVV :  57
at_KAT1    : -LFQGVSR--------NFLFQLVSDIDAEYFPPKEDIILQNEAPTDLYILVSGAVDFTVYVDGHDQ-FQGKAVIGETF :  68
at_KAT2    : -LFHGVSR--------NFLFQLVSDIDAEYFPPREDVILQNEAPTDLYILVSGAVDFTVYVGEEDQ-VQGKAVVGDAF :  68
os_KAT1a   : -LFNGVSG--------NFIAELVMEVQAEYFPPKEDIILQNEGEADVYIVVSGAVNIITTIHGNEQ-VYEKIAEGEMF :  68
os_KAT1b   : -LFKGVSS--------RFIQQLVTEMQAEYFAPKEDIILQNDSPSDLYLLVSGAVDILVFLDGTEQ-VYRRAAEGELL :  68
os_KAT1c   : -LFHGVSF--------TCMIQLVTEMEAEYYPPREVVILQNEAPRDVYILVSGAVEERVEIDGTEK-VQEVLCNGEIF :  68
at_AKT2    : -LFKGVSR--------EILLLLVSKMKAEYIPPREDVIMQNEAPDDVYIIVSGEVEIIDSEMERES-VLGTLRCGDIF :  68
os_AKT2    : -LFKGVSR--------EVLLLMVTKMKPEYIPPKEDVIVQNEAPDDVYIVVSGEVEVIYSDGEAEERVVATLGTRGVF :  69
os_KAT3b   : -LFQGVSD--------SLIVQLVAEMKAEFFPPKANVILENETSTDCYIIISGEVEALTTLADGTEKHVKRIGPRGMA :  69
os_KAT3a   : -LFQGVSD--------KLVLPLVAEMKAESFPPKADIILENEASTDCYIIVSGEVEVLTTLEDGTEKQVMRIGPRGMA :  69
at_KAT3    : -LFKGFPE--------GLLVQLVSQIQAEYFPPKMEIILQNEIPTDFYVIVSGGVDIIASKG-VSEQVLAKLGPGSMA :  68
ec_CRP     : VLGKPQTD--------PTLEWFLSHCHIHKYPSKSTLIHQGEKAETLYYIVKGSVAVLIKDEEGKEMILSYLNQGDFI :  70
               
                                                                                             
             80         *       100         *       120         *       140         *        
at_CNGC16  : CGEELLTWALVPNINH------NLPL----STRTVRTLSEVEAFALRAEDLKFVANQFR-RLHSKKLQHAFRYY : 132
at_CNGC18  : CGEELLTWALMPNSTL------NLPS----STRSVRALSEVEAFALSAEDLKFVAHQFK-RLQSKKLQHAFRYY : 132
os_CNGC18  : CGEELLTWALMPNPSL------NFPQ----STRTVRSVTEVEAFALRAEDLKYVANQFK-RLHSKRLQHAFRYY : 132
os_CNGC16a : AGEELLTWALLPKTNV------HFPL----STRTVQSLTEVEAFALRAEDLKFVANQFR-RLHSKKLQHTF--- : 129
os_CNGC17a : CGEELLGWALVPKPTV------NLPS----STRTVKTIVEVEAFALRAEDLKFVASQFR-RLHSRKLQHTFRYY : 132
os_CNGC17b : CGEELLGWALVPKPTV------NLPS----STRTVKALIEVEAFALQAEDLKFVANQFR-RLHSKRLQHTFRYY : 132
at_CNGC14  : CGEELLAWALLPKSTV------NLPS----STRTVRALEEVEAFALQAGDLKFVANQFR-RLHSKKLQHTFRYY : 132
at_CNGC17  : CGEELLSWALLPKSTL------NLPS----STRTVRALVEVEAFALRAEDLKFVANQFR-RLHSKKLQHTFRFY : 132
os_CNGC16b : CGEELLTWALLPSSRD------SYPS----STRTVKTIAELEAFSLQADDIKCVASTFR-MMHSKHLQHTFRLH : 132
at_CNGC9   : CGEELLTWALDPKSGS------NLPS----STRTAKALTEVEAFALIADELKFVASQFR-RLHSRQVQHTFRFY : 132
at_CNGC6   : CGDELLTWALDPKSGS------NLPS----STRTVKALTEVEAFALIADELKFVASQFR-RLHSRQVQHTFRFY : 132
at_CNGC5   : CGEELLTWALDPKSGV------NLPS----STRTVKALTEVEAFALTSEELKFVASQFR-RLHSRQVQHTFRFY : 132
at_CNGC8   : CGEELLTWALDPKAGS------NLPS----STRTVKALTEVEAFALEAEELKFVASQFR-RLHSRQVQQTFRFY : 132
at_CNGC7   : CGEELLTWALDPKAGS------NLPS----STRTVKALTEVEAFALEAEELKFVASQFR-RLHSRQVQQTFRFY : 132
os_CNGC13  : CGDELLTWALDPKSAA------NFPA----STRTVKALTEVEAFALCAEELKFVASQFR-RLHSRQVQHTFRFY : 132
os_CNGC5c  : CGEELLTWALDPKAGL------SLPS----STRTVRALSEVEAFALHSDELKFVAGQFR-RMHSKQVQHTFRFY : 132
os_CNGC5b  : CGEELLTWALDPKAAA------NLPL----STRTVKAISEVEAFALHADELKFVAGQFR-RLHSKQLQQTFRFY : 132
os_CNGC15  : CGEELLPWALDPRPAA------SLPL----STRTVRAVSEVEAFALVADDLRFVASQFR-RLHSARIRHRFRFY : 132
at_CNGC15  : CGEELLTWALDPRPVV------ILPS----STRTVKAICEVEAFALKAEDLQFVASQFR-RLHTKQLRHKFRFY : 132
at_CNGC3   : CG-DLLTWALDPLSS-------QFPI----SSRTVQALTEVEGFLLSADDLKFVATQYR-RLHSKQLRHMFRFY : 130
at_CNGC11  : CG-DLLTWALYSLSS-------QFPI----SSRTVQALTEVEGFVISADDLKFVATQYR-RLHSKQLQHMFRFY : 130
at_CNGC13  : CGEDLLTWALDPQSSS------HFPI----STRTVQALTEVEAFALAADDLKLVASQFR-RLHSKQLQHTFRFY : 132
at_CNGC10  : CGEDLLPWALDPQSSS------HFPI----STRTVQALTEVEAFALTAEDLKSVASQFR-RLHSKQLQHTF--- : 129
at_CNGC1   : CGEELLTWALDPHSSS------NLPI----STRTVRALMEVEAFALKADDLKFVASQFR-RLHSKQLRHTFRYY : 132
os_CNGC1a  : CGEELLTWALDPTSAS------NLPS----STRTVKTLSEVEAFALRADDLKFVATQFR-RLHSKQLQHTFRFY : 132
os_CNGC1b  : CGEELLTWALDPTSVS------SLPS----STRTVKTMSEVEAFALRAEDLKFVATQFR-RLHSKQLQHTFKFY : 132
at_CNGC12  : CGELLFN-------GS------RLPT----STRTVMTLTEVEGFILLPDDIKFIASHLN-VFQRQKLQRTFRLY : 120
at_CNGC2   : LGDELLSWCLRRPFLD------RLPP----SSATFVCLENIEAFSLGSEDLRYITDHFRYKFANERLKRTARY- : 130
os_CNGC2   : LGDELLSWCLRRPSLD------RLPA----SSATFECVETAQAFCLDAPDLRFITEQFRYKFANEKLKRT---- : 127
os_CNGC4a  : SGDELLSWCMRRPFLE------RLPA----SSSTLVTMESTEAFGLEAADVKYVTQHFRYTFTNDRVRRSARY- : 130
os_CNGC4b  : SGDELLSWCMRRPFME------RLPA----SSSTLVTAESTEAFGLEAGDVKYVTQHFRYTFTSDKVRRSARY- : 130
at_CNGC4   : SGDELLSWCLRRPFVE------RLPP----SSSTLVTLETTEAFGLDAEDVKYVTQHFRYTFVNEKVKRSARY- : 130
os_CNGC19b : CGEELLSWYLEQSSVNRDGGKIKLHGMRLVAIRTVRCLTNVEAFVLRARDLEEVTSQFSRFLRNPLVLGTIRY- : 136
os_CNGC19a : CGEELLTWYLEHSSANRDGGRMRFHGMRLVAIRTV---------------MHSVVS-VAEYLSS-MICMTM--- : 117
at_CNGC19  : CGEELLTWCL--SSINPDGTRIKMPPKGLVSNRNVRCVTNVEAFSLSVADLEDVTSLFSRFLRSHRVQGAIRY- : 134
at_CNGC20  : CGEELLTWCLERSSVNPDGTRIRMPSKGLLSSRNVRCVTNVEAFSLSVADLEDVTSLFSRFLRSHRVQGAIRY- : 136
at_CNTE1   : FGHGIFG---------------DVYS------ADVVAVTELTCLLLMSDHRALLEIKSVSDSDK--ERC-LVED : 117
at_CNTE2   : FGVGLSG---------------NVHS------ADIVAMSQLTCLVLPRDHCHLLETNSIWQSDTSLDKCSLVE- : 119
os_CNTE1   : FGYGTNS---------------SVHQ------VNVIAVSKLTCFVLPNQYGHLLQPKTIWSAEETPENHSLLE- : 121
os_ORK1    : GEIAVLC---------------NIPQ-----PFTVRVCELCRLLRLDKQSFTNILEI-FFVDGRRILSNLS--- : 119
at_SKOR    : GEISILC---------------NIPQ-----PYTVRVAELCRILRLDKQSFMNILEI-FFHDGRRILNNLL--- : 119
at_GORK    : GDISIIC---------------NISQ-----PFTVRVCELCHLLRLDKQSFSNILEI-YFHDGRTILNNIM--- : 119
os_ORK2    : GDVAVIC---------------NIPQ-----PYTVRVCELCSLLRIDKQSLTSILQI-YFKDNSQILSNLL--- : 119
at_AKT5    : GEVGVLC---------------YRPQ-----LFTVRTKRLSQLLRLNRTAFLNLVQA-NVGDGAIIMNNLL--- : 118
at_AKT6    : GEVGVLC---------------YRPQ-----LFTVRTKRLSQLLRLNRTVLLNLVQA-NVGDGAIIMNNLL--- : 118
at_AKT1    : GEIGVLC---------------YRPQ-----LFTVRTKRLCQLLRMNRTTFLNIIQA-NVGDGTIIMNNLL--- : 118
os_AKT1a   : GEIGVLC---------------YRPQ-----LFTVRTRSLCQLLRLNRTAFLSIVQS-NVGDGTIIMNNLI--- : 114
os_AKT1b   : GEIGVLC---------------YRPQ-----LFTARTRSLCQLLRLDRAAFLRIIQS-NIADGTIVMNNLI--- : 107
at_KAT1    : GEVGVLY---------------YRPQ-----PFTVRTTELSQILRISRTSLMSAMHA-HADDGRVIMNNLF--- : 118
at_KAT2    : GEIGVLC---------------YTPQ-----PFTVRTTELSQILRISKKSLMSAMRA-HVEDGRVIMNNLF--- : 118
os_KAT1a   : GEVGSLC---------------NIPQ-----PFTCRTAELSQLLRISKTRLREIIEE-NREDSNILMNNLV--- : 118
os_KAT1b   : GEIGVLC---------------NKPQ-----SFTFRTTKLSQILRISRTKLLGIIQE-NREDGDIIRSNLQ--- : 118
os_KAT1c   : GEIGVIC---------------SIPQ-----PCAFHTIKVSQLLRLNTAVLKNIIKE-NSDDRRVILNNLS--- : 118
at_AKT2    : GEVGALC---------------CRPQ-----SYTFQTKSLSQLLRLKTSFLIETMQI-KQQDNATMLKNFL--- : 118
os_AKT2    : GEVSALS---------------DRPQ-----SFTLRTRTLCQLLRLRQAALKEAMQS-KPEDSVVIIKNFL--- : 119
os_KAT3b   : GEIGVMF---------------SIPQ-----PFTIRSRRLTQVVRISHIHLLQAVRP-NTADGYIVFSNFI--- : 119
os_KAT3a   : GEIGVMF---------------NIPQ-----PFTIRSRKLTQLVRISHSHMVSTIRP-NTADGVVVFSNFV--- : 119
at_KAT3    : GEIGVVF---------------NIPQ-----PFTVRTRRLSQVIRIGHHKFKEMVQSDNDVDAKMIIANF---- : 118
ec_CRP     : GELGLFE---------------EGQE----RSAWVRAKTACEVAEISYKKFRQLIQVNPDILMRLSAQMA---- : 121
 Sequence Alignment for Additional File 1a

                                                                                                 
                    *        20         *        40         *        60         *        80      
os_KAT1b : -----------------------------------------MPRSSRMNLWPHCFPCFDDGDRSGNRFSTVCN-FPDDLL :  38
os_KAT1c : -------------------------------------------------------------------METISNIFHNDPL :  13
os_KAT1a : ---------------------------------------------MTQAHSKSCFHQFWDG----LQIKRSSDSFTVELL :  31
at_KAT1  : ----------------------------------------------MSISWTRNFFERFCVEEYNIDTIKQSSFLSADLL :  34
at_KAT2  : -------------------------------------------------------------------------------- :   -
at_AKT5  : MGIEKRKKMVWFWPEKHEG-------------------------------GVIKEAEDVAAEHISREGTMSHYSFSKGLL :  49
at_AKT6  : --MEKKK--VWFWGVKDDGEGGGGRG-----------------------GGRTKDAEDDVADHLSRDGTMSQYSLSKGLL :  53
at_AKT1  : ---------------MRGG-------------------------------ALLCGQVQDEIEQLSRE--SSHFSLSTGIL :  32
os_AKT1a : --MARWGAARMAACGPWGRNRRVGAGDAFEASEVRRDGRSRMMPACGPWGAGHGGGDPALERELS--RDGSHYSISSAIL :  76
os_AKT1b : --MPTTKCAVPLVSG-----------------------------------AAGGGGSAELTRQLSSTQASPRFSFSSGVL :  43
os_KAT3b : -----------------------------------------------------------------------MAARSELLR :   9
os_KAT3  : ----------------------------------------------------------------------MAASRSELLR :  10
at_KAT3a : MSTTTTEARSPLPLLLRRGRSSTALS--------------------ASTAEARSPLSILQFRRRSSKDVRNITSVSSSLL :  60
at_AKT2  : MDLKYSASHCNLSSDMKLR--------------------------------RFHQHRGKGREEEYDASSLSLNNLSKLIL :  48
os_AKT2  : --MKTSSFESASSSGG--------------------------------------SGGGGGGGGGEGSGSFNLRNLSKLIL :  40
at_GORK  : -------------MG---------------RLRRRQ------EIIDHEEEE------SNDDVSSRRG-KLSLAETFRWLD :  39
at_SKOR  : -------------MGG----------SSGGGVSYRSGGESDVELEDYEVDD------FRDGIVESRGNRFNPLTNFLGLD :  51
os_ORK1  : -------------MGRGIGSKRRVEDDDGENMPGRKKKEEEEEEEDDDGEEEYEVDVVRDRIGSSRGSRLALFGSDLRLG :  67
os_ORK2  : ------------------------------------------MAEEYELNE------IDDTLHGSVGSRLSLFARELKSR :  32
                                                                                                 
                                                                                                 
                    *       100         *       120         *       140         *       160      
os_KAT1b : PSLG----ATAHQPPKLR--KYLVSPYDPRYKVWETFLIILVVYSAWICPLEFAFLR-YLPSAPFVVDDVVNGFFAVDIM : 111
os_KAT1c : PPLG----ARANQSIKLR--KFIISPYDSRYRTWETFLLVLVVYSAWICPFELAYLR-NLSWKVSLVDNIIDSFFAIDII :  86
os_KAT1a : PSLG----ATINHSNKLQ--KFIISPYDPRYRSWELFLIVLVVYSAWICPFELAFLR-DLPSKLLLVENIVDIFFAIDIV : 104
at_KAT1  : PSLG----ARINQSTKLR--KHIISPFNPRYRAWEMWLVLLVIYSAWICPFQFAFIT-YKKDAIFIIDNIVNGFFAIDII : 107
at_KAT2  : -------------------------------------------------------------------------------- :   -
at_AKT5  : PPLGVGATARSSRHIKLR--CFIVSPFDPRYRAWDWFLVILVLYTAWASPFEFGFLQ-TPRAPLSILDNVVNGFFAVDIV : 126
at_AKT6  : PSL--GANNRSSRDVILP--RFIVSPFDPRYRAWETFLVFLVLYTAWASPFEFGFLQ-KPRPPLSILDNIVNGFFAVDIV : 128
at_AKT1  : PSLG----ARSNRRVKLR--RFVVSPYDHKYRIWEAFLVVLVVYTAWVSPFEFGFLR-KPRPPLSITDNIVNAFFAIDII : 105
os_AKT1a : PSLG--ARS--NRRIKLR--RFIISPYDRRYRIWETFLIVLVVYSAWVSPFEFGFIP-KPTGALATADNVVNAFFAVDII : 149
os_AKT1b : PSLG--SRGGGERHARLR--RFIVSPYDRRYELWNNYLILLVVYSAWVTPFEFGFVP-EPAGALAAADNAVNAFFAVDIV : 118
os_KAT3b : PAFG-------EASPSLG--RFVINPHSCSYRWWHMFLIMLVLYSAWASPFELSMEK-AASIALVVTDLVVDVFFAIDIA :  79
os_KAT3  : PAFG-------EPSPSLG--PFVVNPHTCSYRWWQKFLIVLVLYTAWASPFELAMEK-SASAALAVTELVVDAFFAVDIA :  80
at_KAT3a : PAFGT---FIEDDNPSSK--PFIVLHFDRRYRLWELFLVILVGYSAWASLFELAFEK-AAEGALLTIDLVVDFFFAVDII : 134
at_AKT2  : PPLGVASYNQNHIRSSG----WIISPMDSRYRCWEFYMVLLVAYSAWVYPFEVAFLNSSPKRNLCIADNIVDLFFAVDIV : 124
os_AKT2  : PPLGVPAGGHAQSGHAGPNDRRVISPLDSRYRCWDTFMVVLVAYSAWVYPFEVAFMNASPKGGLEVADIVVDLFFAVDIV : 120
at_GORK  : ---SSEHRRIETDGHNDYK--YIIHPKNRWYKAWEMFILVWAIYSSLFTPMEFGFFR-GLPERLFVLDIVGQIAFLVDIV : 113
at_SKOR  : FAGGSGGKFTVINGIRDISRGSIVHPDNRWYKAWTMFILIWALYSSFFTPLEFGFFR-GLPENLFILDIAGQIAFLVDIV : 130
os_ORK1  : RFRPRRRRVAPVDGDDGIFQDFVIDPDNKWYRLWTRFILVWAVYSSFFTPLEFGFFR-GLPRNLFFLDIAGQIAFLIDIV : 146
os_ORK2  : R-SSSWHGGTALRLPKDLYESLVIHPNGRWYRIWANMMFLWSIYSTFFTPFEFSFFR-GLPDQLLDLECV-QLVFLADVA : 109
 
                                                                                                 
                    *       180         *       200         *       220         *       240      
os_KAT1b : LTFFVPFVDKKSYLLVNDPKKIAVRYLSS-WFVFDVCSTVPFHSISLLFNEHGHDLGFK-FLNVLRLWRLRRVSSMFARL : 189
os_KAT1c : LTFFLAYLDQKSYLLVDDPKRIVARLLS-----------------------------------MLRLWRLRRLSELFARL : 131
os_KAT1a : LTFFVAYVDSKTHLLVDDRKRIAMRNPTF-G--------IEKGPNDSALTLANYDHLQH-HFNQSSFYLHTRGMTLLSRL : 174
at_KAT1  : LTFFVAYLDSHSYLLVDSPKKIAIRYLST-WFAFDVCSTAPFQPLSLLFNYNGSELGFR-ILSMLRLWRLRRVSSLFARL : 185
at_KAT2  : -------------------------YLST-WFAFDVCSTAPFQSLSLLFKYNGSEIGFR-VLSMLRLWRLRRVSSLFARL :  53
at_AKT5  : LTFFVAFLDKATYLLVDDPKRIAWRYTST-WLIFDVVSTVPYELFGSLLHNTIQGYG---IFSMLRLWRLHRVSKCFARL : 202
at_AKT6  : LTFFVAFLDKVTYLLVDDPKRIAWRYAST-WLIFDVVSTFPYEIFGSLLHESIQGYG---IFSMLRLWRLRRVSNCFARL : 204
at_AKT1  : MTFFVGYLDKSTYLIVDDRKQIAFKYLRS-WFLLDLVSTIPSEAA---MRISSQSYG---LFNMLRLWRLRRVGALFARL : 178
os_AKT1a : LTFFVAYLDKMSYMLEDDPKKIAWRYSTT-WLVLDVASTIPSEFARRILPSKLRSYG---FFNMLRLWRLRRVSSLFSRL : 225
os_AKT1b : LTFFVAYTDPKTFLLQDDPRKIALRYITT-WFVLDVVATIPTELARRILPPDLRSYG---FFGILRLWRLHRVGILFARL : 194
os_KAT3b : LSFFVAYRDTSTGLLITDRRKITMRYLKRPCFALDVASTIPLQIIYQLVTG--KRQGLWGLLNLLRLWRLRRVSKLFARV : 157
os_KAT3  : VSFFVAYRDASTGLLVTDRKKIATRHLARPCLALDVASTIPLQMIYRIVSG--KRQALYGLLNLLRLWRLRRVSKLFARL : 158
at_KAT3a : LTFFVSYLDNTTYLNVTDHKLIAKRYLKSVAFVMDVASTLPIQFIYKTITGDVGRGQAFGFLNLLRLWRLRRVAELFKRL : 214
at_AKT2  : LTFFVAYIDERTQLLVREPKQIAVRYLST-WFLMDVASTIPFDAIGYLITGTSTLNITCNLLGLLRFWRLRRVKHLFTRL : 203
os_AKT2  : LTFFVAYIDSRTQLLVRDRRRIATRYLST-FFIMDVASTIPFQGLAYIVTGEVRESPAFSLLGILRLWRLRKVKQFFTRL : 199
at_GORK  : LQFFVAYRDTQSYRTVYKPTRIAFRYLKS-HFLMDFIGCFPWDLIYKASGKHELVR----YLLWIRLFRVRKVVEFFQRL : 188
at_SKOR  : LTFFVAYRDSRTYRMIYKRSSIALRYLKS-TFIIDLLACMPWDIIYKAAGEKEEVR----YLLLIRLYRVHRVILFFHKM : 205
os_ORK1  : LRFFVAYRDPDTYRMVHNPTSIALRYCKS-SFIFDLLGCFPWDAIYKACGSKEEVR----YLLWIRLTRAMKVTEFFRSM : 221
os_ORK2  : VHFFLAYRDPHTYRMVHDKRHIALRYIKG-SFALDVLGCFPWDAIYKVTGRVEAVR----WLVWVRLYRGRKVMAFFKRV : 184

                                                                                                 
                    *       260         *       280         *       300         *       320      
os_KAT1b : EKDIRFNYAVIRCTKLISVTLFAIHCAGCINYLIADRYPDPRR--TWIGAVMPN------FREDGLWIRYVTAMYWSITT : 261
os_KAT1c : EKDIRLNYYWIRCTKLISVTLFAVHCSGCFNYLIADRYPNPAR--TWIGAAIPN------YRSQNLWVRYVTAIYWSITT : 203
os_KAT1a : EKDIRFNYFWTRCSKLISVTLFAVHCAGCFNYMIADRYPNPEK--TWIGAVMST------FRSESLWTRYITALYWSITT : 246
at_KAT1  : EKDIRFNYFWIRCTKLISVTLFAIHCAGCFNYLIADRYPNPRK--TWIGAVYPN------FKEASLWNRYVTALYWSITT : 257
at_KAT2  : EKDIRFNYFWTRCTKLISVTLFAVHCAGCFAYLIADQYHDPTK--TWIGAVYPN------FKETSVWSRYVTALYWSITT : 125
at_AKT5  : EKDRKYNYFWIRCTKLLLVSLFVVHCGACFCYSIAAHYPDPSM--TFMALAEAN------WKQKSLLIRYVTAMYWSITT : 274
at_AKT6  : EKDRKYSYFWVRCSKLLLVTLFVIHCGACFLYSIAAHYPDPSK--TFMALTDEN------WKESPIAVRYNTAMYWSITT : 276
at_AKT1  : EKDRNFNYFWVRCAKLVCVTLFAVHCAACFYYLIAARNSNPAK--TWIGANVAN------FLEESLWMRYVTSMYWSITT : 250
os_AKT1a : EKDRHFNYFWVRCAKLICVTLFAVHCAACFYYLLADRYPVPTS--TWIGNYMAD------FHERSLWIRYVTSVYWSITT : 297
os_AKT1b : EKDRKFSYFWVRCVKLVCVTLFAVHCSACFYYLLADRYPDPTN--TWISAYMPN------FHKASIWSRYVASMYWSITT : 266
os_KAT3b : EKDIRFNYLWTRLIKLLCVTLFALHFAACIYLWMAFNYKIKEL--TWIGSQIHS------FEDRSVWFCYTCAVYWSITT : 229
os_KAT3  : EKDIRFSYLWTRLIKLLYVTLFAVHFASCIYLWMAFHHKAKEL--TWIGSQFHG------FEDRSVWFCYTCAVYWSITT : 230
at_KAT3a : EKDAHFNYFVIRVIKLLCVTIFWIHLAGCILYWIAYHYPRPTD--TWIGSQVED------FKERSVWLGYTYSMYWSIVT : 286
at_AKT2  : EKDIRYSYFWIRCFRLLSVTLFLVHCAGCSYYLIADRYPHQGK--TWT-DAIPN------FTETSLSIRYIAAIYWSITT : 274
os_AKT2  : EKDIRFNYFWIRCARLIAVTLFLVHCAGCLYYLIADRYPHREK--TWIGAVIPD------FQEASLWIRYTSSVYWSITT : 271
at_GORK  : EKDTRINYLFTRILKLLFVEVYCTHTAACIFYYLATTLPPEQEGYTWIGSLKLGDYSYENFREIDLWKRYTTALYFAIVT : 268
at_SKOR  : EKDIRINYLFTRIVKLIFVELYCTHTAACIFYYLATTLPASQEGYTWIGSLKLGDYSYSKFREIDLWTRYTTSMYFAVVT : 285
os_ORK1  : EKDIRINYLFTRIVKLIVVELYCTHTAACIFYYLATTLPESMEGYTWIGSLQLGDYSYSHFREIDLTKRYMTSLYFAIVT : 301
os_ORK2  : EKDIRVSYLLTRIVKLITVELYCTHTAACGFYYLATTLPPAREGGTWIGSLSLGDARYINFREVDLLTRYVTSLYLAIVT : 264
           
                                                                                                 
                    *       340         *       360         *       380         *       400      
os_KAT1b : LTTTG---YGDLHAENAREMLFGICYMLFNLWLTAYLIGNMTNLVVHSTSRTRDFRDVVQAASEFAARNQLPQQIEEQML : 338
os_KAT1c : LTTTG---YGDLHAENQREMLFSICYMLFNLGLTAYLIGNMTNLVVQGSCRTRNFRDTIHAASQFAARNQLPGHIKDEML : 280
os_KAT1a : LTTTG---YGDLHAENPTEMLFDIVYMMFNLGLTAYLIGNMTNLVVHGTSRTRKFRDSIQAASEFAARNQLPENIKQQVL : 323
at_KAT1  : LTTTG---YGDFHAENPREMLFDIFFMMFNLGLTAYLIGNMTNLVVHWTSRTRTFRDSVRAASEFASRNQLPHDIQDQML : 334
at_KAT2  : LTTTG---YGDLHAENPREMLFFVFFMLFNLGFTSYLIGNMTNLVVHWTSRTRNFRDTVRAASEFASRNQLPPNIQDQML : 202
at_AKT5  : FSTTG---YGDIHGNNAEERAFILFYMIFNLGLLAYIIGNMTNLVVHVTSRTRNFRDTIQAASAFAQRNNLPLGLQEQMV : 351
at_AKT6  : FSTTG---YGDIHGVNSREMTFILFYMVFNLGLSAYIIGNMTNLVVHVTGRTRKFRDTIQAASGFGQRNNLPVRLQDQMV : 353
at_AKT1  : LTTVG---YGDLHPVNTKEMIFDIFYMLFNLGLTAYLIGNMTNLVVHGTSRTRNFRDTIQAASNFAHRNHLPPRLQDQML : 327
os_AKT1a : LTTVG---YGDLHAENTREMIFNIFYMLFNLGLTAYLIGNMTNLVVHGTSRTRNYRDTIQAATSFGVRNQLPPRLQDQMI : 374
os_AKT1b : LSTVG---YGDMHAENTGEMVFTTTYMLFNLGLTAYIIGNMTNLVVHGTSRTRKFRDMIQAATSFAQRHQLPARLQEQMV : 343
os_KAT3b : LATVG---YGDLHATNIGEMLFSIAFMLFNMGLTSYIIGNITNLVVRETSNTFKMRDMVQRVSEFGRMNRLPEAMREQML : 306
os_KAT3  : LATVG---YGDLHAANTGEMLFSIAFMLFNMGLTSYIIGNITNLVVHETTNTFKMRDMVQRTSVFGRTNRLPVAMREQMM : 307
at_KAT3a : LTTVG---YGDLHAVNSREKTFNMFYMLFNIGLTSYIIGIMTNLVVHGALRTFAMRSAINDILRYTSKNRLPDTMREQML : 363
at_AKT2  : MTTVG---YGDLHASNTIEMVFITVYMLFNLGLTAYLIGNMTNLVVEGTRRTMEFRNSIEAASNFVNRNRLPPRLKDQIL : 351
os_AKT2  : MTTVG---YGDMHAQNTVEMIFNIFYMLFNLGLTAYLIGNMTNLVVEGTRRTMEFRNSIRAASNFVGRNHLPPRLKQQIL : 348
at_GORK  : MATVG---YGDIHAVNLREMIFVMIYVSFDMVLGAYLIGNVTALIVKGS-NTERFRDKMNDLISFMNRKKLGRDLRSQIT : 344
at_SKOR  : MATVG---YGDIHAVNMREMIFAMVYISFDMILGAYLIGNMTALIVKGS-KTERFRDKMADIMRYMNRNKLGRNIRGQIT : 361
os_ORK1  : MATVG---YGDIHAVNVREMIFIMIYVSFDMILGAYLIGNMTALIVKGS-RTERFRDKMKEVIRYMNRNKLGKDIREQIK : 377
os_ORK2  : MATVGGSGYGDIHAVNTREMAFTVVYISFSIVLSAYLIGNMTALIVKGS-RTERFRDRMTDLIRYMNRNRLGSAIRSQVK : 343
             
                                                                                                 
                    *       420         *       440         *       460         *       480      
os_KAT1b : NHICLRYKT--DGLKQQETLDVLPKAMRSSISHYLFFRVVQGAYLFKGVSSRFIQQLV-------------------TEM : 397
os_KAT1c : SHICLRYKT--EGLKQKETLDSLPKGIRSSIACNLFLPVIEKVYLFHGVSFTCMIQLV-------------------TEM : 339
os_KAT1a : SHFCLQFKT--EGLNQQVMLDCLPKGIRSSIAYSLFFPIIRQAYLFNGVSGNFIAELV-------------------MEV : 382
at_KAT1  : SHICLKFKT--EGLKQQETLNNLPKAIRSSIANYLFFPIVHNIYLFQGVSRNFLFQLV-------------------SDI : 393
at_KAT2  : SHICLKFKT--EGLKQQEALNGLPKRIRSSIANYLFFPIVQNVYLFHGVSRNFLFQLV-------------------SDI : 261
at_AKT5  : AHLSLRYRTDSEGLQQQEIIDSLPKAIRSSISHYLFYEVVDKTYLFHGISNDLLFQLV-------------------SEM : 412
at_AKT6  : AHLCLRYRTDSEGLQQQEIIDSLPKAIRSSISHYLFYEVVDKIYLFHGISNDLLFQLV-------------------TEM : 414
at_AKT1  : AHLCLKYRTDSEGLQQQETLDALPKAIRSSISHFLFYSLMDKVYLFRGVSNDLLFQLV-------------------SEM : 388
os_AKT1a : SHISLKYRTDSEGLQQQEILDSLPKAIKSSISQYLFFHLVQNVYLFQGVSNDLIFQLLRTVTEYAYLVLLLDFVFEVSEM : 454
os_AKT1b : SHLSLKFRTNSEGLHQQETFEALPKAIKSSISHHLFFGLVQNVYLFEGVSNDLIFQLV-------------------SEM : 404
os_KAT3b : ASVQLRFRT--DEQLQQEMLSELPKAVRSGVMKHMFKSAIESCYLFQGVSDSLIVQLV-------------------AEM : 365
os_KAT3  : ESLQLRFRA--EEQLQQEMLSELPKAVRSGIAQHMFRGAVQSCYLFQGVSDKLVLPLV-------------------AEM : 366
at_KAT3a : AHMQLKFKT--AELRQEEVLQDLPKAIRSSINQHLFRSIIEEAYLFKGFPEGLLVQLV-------------------SQI : 422
at_AKT2  : AYMCLRFKA--ESLNQQHLIDQLPKSIYKSICQHLFLPSVEKVYLFKGVSREILLLLV-------------------SKM : 410
os_AKT2  : AYMCLKFRA--ESLNQQQLMDQLPKSICKGICEYLFLPVVKDVYLFKGVSREVLLLMV-------------------TKM : 407
at_GORK  : GHVRLQYDS--HYT-DTVMLQDIPASIRAKIAQLLYLPYIKKVPLFKGCSTEFINQIV-------------------IRL : 402
at_SKOR  : GHLRLQYES--SYT-EAAVLQDIPVSIRAKIAQTLYLPYIEKVPLFRGCSSEFINQIV-------------------IRL : 419
os_ORK1  : GHLRLQYES--SYT-EASVLQDIPVSIRAKISQTLYKPYIESIPLFKGCSAEFIQQIV-------------------IRL : 435
os_ORK2  : DHLMLQYES--SYTRDRVIVDDIPVAVRSKMSQTLYLDMVSRVGLFRGCSDDFLSQIV-------------------LKL : 402
6      
                                                                                                 
                    *       500         *       520         *       540         *       560      
os_KAT1b : QAEYFAPKEDIILQNDSPSDLYLLVSGAVDILVFLD-GTEQVYRRAAEGELLGEIGVLCNKPQSFTFRTTKLSQILRISR : 476
os_KAT1c : EAEYYPPREVVILQNEAPRDVYILVSGAVEERVEID-GTEKVQEVLCNGEIFGEIGVICSIPQPCAFHTIKVSQLLRLNT : 418
os_KAT1a : QAEYFPPKEDIILQNEGEADVYIVVSGAVNIITTIH-GNEQVYEKIAEGEMFGEVGSLCNIPQPFTCRTAELSQLLRISK : 461
at_KAT1  : DAEYFPPKEDIILQNEAPTDLYILVSGAVDFTVYVD-GHDQFQGKAVIGETFGEVGVLYYRPQPFTVRTTELSQILRISR : 472
at_KAT2  : DAEYFPPREDVILQNEAPTDLYILVSGAVDFTVYVG-EEDQVQGKAVVGDAFGEIGVLCYTPQPFTVRTTELSQILRISK : 340
at_AKT5  : KAEYFPPKEDVILRNEAPSDFYIMVTGAVDIIARVN-GVDQVVGEAQTGHVFGEVGVLCYRPQLFTVRTKRLSQLLRLNR : 491
at_AKT6  : KAEYFPPKEDVILQNEAPTDFYILVTGAVDIIARVN-GVEQVVSEAQRGHVFGEVGVLCYRPQLFTVRTKRLSQLLRLNR : 493
at_AKT1  : KAEYFPPKEDVILQNEAPTDFYILVNGTADLVDVDT-GTESIVREVKAGDIIGEIGVLCYRPQLFTVRTKRLCQLLRMNR : 467
os_AKT1a : KAEYFPPREDVILQNEAPTDFYILVSG------------SVVIQVATSGEVVGEIGVLCYRPQLFTVRTRSLCQLLRLNR : 522
os_AKT1b : NAEYFAPREDIILQNEAPADFYIIVSG------------SMLAGMAKSGDVVGEIGVLCYRPQLFTARTRSLCQLLRLDR : 472
os_KAT3b : KAEFFPPKANVILENETSTDCYIIISGEVEALTTLADGTEKHVKRIGPRGMAGEIGVMFSIPQPFTIRSRRLTQVVRISH : 445
os_KAT3  : KAESFPPKADIILENEASTDCYIIVSGEVEVLTTLEDGTEKQVMRIGPRGMAGEIGVMFNIPQPFTIRSRKLTQLVRISH : 446
at_KAT3a : QAEYFPPKMEIILQNEIPTDFYVIVSGGVDIIASKG-VSEQVLAKLGPGSMAGEIGVVFNIPQPFTVRTRRLSQVIRIGH : 501
at_AKT2  : KAEYIPPREDVIMQNEAPDDVYIIVSGEVEIIDSEMERES-VLGTLRCGDIFGEVGALCCRPQSYTFQTKSLSQLLRLKT : 489
os_AKT2  : KPEYIPPKEDVIVQNEAPDDVYIVVSGEVEVIYSDGEAEERVVATLGTRGVFGEVSALSDRPQSFTLRTRTLCQLLRLRQ : 487
at_GORK  : HEEYFLPGEVITEQGNVVDHLYFVCEGLLEALVTKTDGSEESVTLLGPHTSFGDISIICNISQPFTVRVCELCHLLRLDK : 482
at_SKOR  : HEEFFLPGEVIMEQGSVVDQLYFVCHGVLEEIGITKDGSEEIVAVLQPDHSFGEISILCNIPQPYTVRVAELCRILRLDK : 499
os_ORK1  : QEEFFLPGEVILEQGSAVDQLYFVCHGALEGVGIGEDGQEETILMLEPESSFGEIAVLCNIPQPFTVRVCELCRLLRLDK : 515
os_ORK2  : HEEFFLPGEVILEQGTVVDQIYIVAHGCLEEVANGEDGSEEIISELRPYGIVGDVAVICNIPQPYTVRVCELCSLLRIDK : 482
 
                                                                                                 
                    *       580         *       600         *       620         *       640      
os_KAT1b : TKLLGIIQE-NREDGDIIRSNLQQVNV----------------------------------------------------- : 502
os_KAT1c : AVLKNIIKE-NSDDRRVILNNLSQKMNQDHRFSTEV---MEKSLQMMHQHFGEYNRCSALNQDNEKNELKANNGHSMALE : 494
os_KAT1a : TRLREIIEE-NREDSNILMNNLVQKLKLRESLP-----------DMNQPDRRFLSKYELFHVPREAWLLKKSQLH----- : 524
at_KAT1  : TSLMSAMHA-HADDGRVIMNNLFMKLRG-------------------QQSIAIDDSNTSG-HENRDFKSMGWE------- : 524
at_KAT2  : KSLMSAMRA-HVEDGRVIMNNLFMKLRG-------------------QQSIAIDDPNSEPESLLKEWLVGGSK------- : 393
at_AKT5  : TAFLNLVQA-NVGDGAIIMNNLLQHLKDSTD-PVMKGILAETELMLAQGKMDLPLSLCFAAARGDDLLLHQLLKRGSN-- : 567
at_AKT6  : TVLLNLVQA-NVGDGAIIMNNLLQHLKDSED-PVMKGVLADTEHMLAQGKMDLPLSLCFAAARGDDLLLHQLLRRGSS-- : 569
at_AKT1  : TTFLNIIQA-NVGDGTIIMNNLLQHLKEMND-PVMTNVLLEIENMLARGKMDLPLNLCFAAIREDDLLLHQLLKRGLD-- : 543
os_AKT1a : TAFLSIVQS-NVGDGTIIMNNLIQFLKEQKENSVMAGVVKEIESMLARGNLDLPITLCFAVTRGDDFLLHQLLKRGMD-- : 599
os_AKT1b : AAFLRIIQS-NIADGTIVMNNLIQYLREKKEIASIVAVAKEIDDMLARGQMDFPITLCFAASKGDSFLLHQLLKRGLD-- : 549
os_KAT3b : IHLLQAVRP-NTADGYIVFSNFIQYLES---------------------------------------------------- : 472
os_KAT3  : SHMVSTIRP-NTADGVVVFSNFVLYLES---------------------------------------------------- : 473
at_KAT3a : HKFKEMVQSDNDVDAKMIIANFMTYLKG---------------------------------------------------- : 529
at_AKT2  : SFLIETMQI-KQQDNATMLKNFLQHHKKLSNLDIGDLKAQQNGENTDVVPPNIASNLIAVVTTGNAALLDELLKAKLS-- : 566
os_AKT2  : AALKEAMQS-KPEDSVVIIKNFLKHQIEMHDMKVEDLLGEDAAGEYDHG--NIPCNLLTVAATGNSSFLEDLLKVGMD-- : 562
at_GORK  : QSFSNILEI-YFHDGRTILNNIMEEKESNDR---IKKLESDIVIHIGKQEAELALKVNSAAFQGDFYQLKSLIRSGAD-- : 556
at_SKOR  : QSFMNILEI-FFHDGRRILNNLLEGKESNVR---IKQLESDITFHISKQEADVALKLNSAAFYGDLYQLKSLIRAGGD-- : 573
os_ORK1  : QSFTNILEI-FFVDGRRILSNLSESSEYGSR---IKQLESDITFHIGKQEAELTLRVNNAAFYGDMHQLKSLIRAGAD-- : 589
os_ORK2  : QSLTSILQI-YFKDNSQILSNLLKGKETESK---RKQLESDITYLLAKQESELVLGVNNAAYHGDIFRLKSLISAGAD-- : 556
 
                                                                                                 
                    *       660         *       680         *       700         *       720      
os_KAT1b : -------------------------------------------------------------------------------- :   -
os_KAT1c : WKRVTIHMYSQRNKRPEAPLAKVINLPGSLDKLFAIACQKFNNYRLTKLVNPEFAEIDDITVIRDGLGEFRLVNIYTRKS : 574
os_KAT1a : --------YTEHTSRDSSNNTPVFGGDRYSRQLLGEATRSSASENENSSMTDKEENHDEVHTN---------CEIKKRTE : 587
at_KAT1  : ---------EWRDSRKD-GYGLDVTNPT-SDTALMDAIHKEDTEMV----KKILKEQ----------------KIERAKV : 573
at_KAT2  : ---------TGEGNASDQGHGHKYLQLHDSENIDMGSTEWRDSRRSGYGETKRVREH----------------TIEIEEG : 448
at_AKT5  : --------PNETDKNGRTALHIAASKGSQYCVVLLLEHGADPNIRDSEGSVPLWEAIIGRHEENAKLLSENGATLSFDTV : 639
at_AKT6  : --------PNEMDKDGRTALHIAASKGSHYCVVLLLEHGADPNIRDSEGNVPLWEAIIGRHREIAKLLAENGAKLSLDSV : 641
at_AKT1  : --------PNESDNNGRTPLHIAASKGTLNCVLLLLEYHADPNCRDAEGSVPLWEAMVEGHEKVVKVLLEHGSTIDAGDV : 615
os_AKT1a : --------PNESDNDGHTALHIAASKGNEQCVRLLLEYGADPNARDSEGKVPLWEALCEKHAAVVQLLVEGGADLSSGDT : 671
os_AKT1b : --------PNESDHYGRTALHIAASNGNEQCVRLLLENGADSNSRDPEGRVPLWEALCRRHQTVVQLLVDAGADLSGGDA : 621
os_KAT3b : ---------LKVQTK-DVAFVSDHLWNGNSMVLRRATEVAVDESKEAAHKMLPCKE------------------------ : 518
os_KAT3  : ---------LKVKAK-ETAFVRDHLRNGYSTVLGSATMFDVDESKESAHKMLPCKE------------------------ : 519
at_KAT3a : ---------LNDELKKEIPFLRDLLDDADAQVQETVQSEETPQSNDEEIVTVSRHEN----------------------- : 577
at_AKT2  : --------PDITDSKGKTPLHVAASRGYEDCVLVLLKHGCNIHIRDVNGNSALWEAIISKHYEIFRILYHFAAISDP-HI : 637
os_AKT2  : --------PDVGDSKGRTALHIAASKGYEDCVLVLLKQACNVNIKDAQGNTALWNAIAARHHKIFNILYHFARVSSPHHA : 634
at_GORK  : --------PNKTDYDGRSPLHLAACRGYEDITLFLIQEGVDVNLKDKFGHTPLFEAVKAGQEGVIGLLVKEGASFNLEDS : 628
at_SKOR  : --------PNKTDYDGRSPLHLAASRGYEDITLYLIQESVDVNIKDKLGSTPLLEAIKNGNDRVAALLVKEGATLNIENA : 645
os_ORK1  : --------PKNTDYDGRSPLHLAACKGFEDVVQFLLHEGVDIDLSDKFGNTPLLEAVKQGHDRVATLLFSKGAKLSLENA : 661
os_ORK2  : --------PSKSDYDGRTALHIAALRGYENIVRFLIQRGANVNSIDRFGNSPLLQAVKSGHDRITSLLVEHGAILNLEDA : 628
                                                                                                 
                                                                                                 
                    *       740         *       760         *       780         *       800      
os_KAT1b : -------------------------------------------------------------------------------- :   -
os_KAT1c : QQQFILNVSKLYQFDIGTRQGHGAMWHAAAGGGDDEGDGGARRPMHGVPNKETTASRGSLKVEEKGMDVVVVEGIQEAIV : 654
os_KAT1a : EHCIQINS-------------------------EDSSSTYSQRTMN-----ATVQTGSPHKTEEN---------ITRRIP : 628
at_KAT1  : ERS------------------------------SSETAGR--------SYANDSSKKDPYCSSSN------------QII : 603
at_KAT2  : EKP------------------------------NKEFDGKGCSDADLTSFEFHSQEAYPYCSSNI------------QIK : 486
at_AKT5  : GYFSCLAVGQNNLNALKDIVKYGGDISLSDV-NGTTALHRAVSEGNLEIVQFLLEKGADMDKPDVYGWTARALAEHQGHE : 718
at_AKT6  : SYFSGLAVEKNCLDALKDIIKYGGDVTLPDG-NGTTALHRAVSEGHLEIVKFLLDQGADLDWPDSYGWTPRGLADHQGNE : 720
at_AKT1  : GHFACTAAEQGNLKLLKEIVLHGGDVTRPRA-TGTSALHTAVCEENIEMVKYLLEQGADVNKQDMHGWTPRDLAEQQGHE : 694
os_AKT1a : GLYACIAVEESDTELLNDIIHYGGDVNRARR-DGTTALHRAVCDGNVQMAELLLEHGADIDKQDGNGWTPRALAEQQGHD : 750
os_AKT1b : APYARVAVEQNDAALLGEIVRHGGDVSGACSGDGTTALHRAVLDGNVQMARLLLEHGADADAEDVNGLTPRAVAEQGGHA : 701
os_KAT3b : ------------------------------------------------PKRVVIHEQLPNATSTA--------------- : 535
os_KAT3  : ------------------------------------------------PKRVSIHEHLLNGTGTA--------------- : 536
at_KAT3a : -------------------------------------GQIEERRREGVPKRVIIHGQAPPNQDNK--------------- : 605
at_AKT2  : AGDLLCEAAKQNNVEVMKALLKQGLNVDTEDHHGVTALQVAMAEDQMDMVNLLATNGADVVCVNTHN-----------EF : 706
os_AKT2  : AGDLLCLAARRGDLDTLRELLKHGLAVDSEDRDGATALRVALAEGHADVARLLVLNGASVDRAASHNEQQAAAAVSVDEL : 714
at_GORK  : GNFLCTTVAKGDSDFLKRLLSSGMN-PNSEDYDHRTPLHVAASEGLFLMAKMLVEAGASVISKDRWGNSPLDEARLCGNK : 707
at_SKOR  : GTFLCTVVAKGDSDFLKRLLSNGID-PNSKDYDHRTPLHVAASEGFYVLAIQLVEASANVLAKDRWGNTPLDEALGCGNK : 724
os_ORK1  : GSHLCTAVARGDTDFVRRALAYGGD-PNARDYDHRAPLHIAAAEGLYLMAKLLVDAGASVFATDRWGTTPLDEGRRCGSR : 740
os_ORK2  : GGYLCRVVRGGRIDLLKKLLRFGIS-PNCRNYDQRTPLHIAAAEGLHLVASTLIESGADIQAKDRWGNTPLDEGRRCSSK : 707
                                                                                                 
                                                                                                 
                    *       820         *       840         *       860         *       880      
os_KAT1b : -------------------------------------------------------------------------------- :   -
os_KAT1c : RTRTTPRP------------------------------------------------------------------------ : 662
os_KAT1a : DEYYIKEA------------------------------------------------------------------------ : 636
at_KAT1  : K--PCKRE------------------------------------------------------------------------ : 609
at_KAT2  : QHEAAKPK------------------------------------------------------------------------ : 494
at_AKT5  : DIKALFYN-----QRPVERKTILVS-GTP----EIKPLMKHSSEPVMTH-HHSREAMPPLARAVSQRRKLSNFKNSLFGI : 787
at_AKT6  : EIKTLFHN-----HRPVEKKPKPIP-GIPQSPVTGKPLMKYSSEPTM---HSGELVLDGGQVVVSQKRKLNNFRNSLFGI : 791
at_AKT1  : DIKALFRE-----KLHERRVHIETSSSVPILKTGIRFLGRFTSEPNIRP-ASREVSFR--IRETRARRKTNNFDNSLFGI : 766
os_AKT1a : DIQLLFRSRKAATASGHHHVPSSTTTRVAPAAAAASLIGRFNSEPMMKNMIHEDADLPSRVLPEKLRRKRVTFQNSLFGV : 830
os_AKT1b : DMQLAFAS--ATRHEPRKARPPPPASAIVPVPLRDGVDSSPSS-SSRRGRTSSTSAASARSTPQRMAN----FRNSLFGV : 774
os_KAT3b : -------------------------------------------------------------------------------- :   -
os_KAT3  : -------------------------------------------------------------------------------- :   -
at_KAT3a : -------------------------------------------------------------------------------- :   -
at_AKT2  : TPLEK----------------------LRVVEEEEEEERGR--------------------------------------- : 725
os_AKT2  : RELMKTRELAHPVTIVVDSPSPAAAAVIREVGSSGDSRNGRRQSARSDG------------------------------- : 763
at_GORK  : KLIKLLEDVKNAQSSIYPSSLRELQEERIERR------------------------------------------------ : 739
at_SKOR  : MLIKLLEDAKNSQISSFPSGSKEPKD-KVYKK------------------------------------------------ : 755
os_ORK1  : TMVQLLEAAKSGELSRYPERGEEVRD-KMHPR------------------------------------------------ : 771
os_ORK2  : PLVRILEQARTVATN----------------------------------------------------------------- : 722
                                                                                                 
                                                                                                 
                    *       900         *       920         *       940         *       960      
os_KAT1b : -------------------------------------------------------------------------------- :   -
os_KAT1c : --------------------------------QGQGRLYRSWSQGEDDSTSHRALQSPPPSRIFRDFDRNQINFPIETQF : 710
os_KAT1a : --------------------------------NKRVTIHKYRHNSTVSAAQNGKLIKLPTS--------LEELFKIGSQK : 676
at_KAT1  : --------------------------------EKRVTIHMM-----SESKN-GKLILLPSS--------IEELLRLASEK : 643
at_KAT2  : --------------------------------DKRVTIHLK-----SRDKDLSKLIILPAS--------IEELLRLAGEK : 529
at_AKT5  : MSAAKTG------DEGGASTRTGISEGVGG--VYPARVTISG-----EASSSGKVVKLPDS--------LEELIEIGEKK : 846
at_AKT6  : ISAANSA------DDGGEVPRSPAVPGGGGSMIYPERVTISSPE---NGETGGKVVLLPNS--------MEELLKIGENK : 854
at_AKT1  : LANQSVP------KNG----LATVDEGRTG---NPVRVTISCAE---KDDIAGKLVLLPGS--------FKELLELGSNK : 822
os_AKT1a : ISSSQAQRETDHPLSRGGLAATGSPNPSSGSRNAVIRVTISCPE-KG--NTAGKLVLLPQT--------LDMLLELGAKK : 899
os_AKT1b : ISSSHAFHHEGGYRGGGGGGGAAAERERSSSSPPLVRVAISCPESRGGKDHSSKLVFMPET--------LRGLLELGAAR : 846
os_KAT3b : ----------------------------------------------LHP-SPGKLVLLPDS--------MQELMKLSEKK : 560
os_KAT3  : ----------------------------------------------LNG-SSGKLVILPDS--------MQDLMKLSEKK : 561
at_KAT3a : ----------------------------------------------NNGDSNGRLIILPDS--------IQLLFDLAEKK : 631
at_AKT2  : ---------------------------------VSIYRGHPLERRERSCNEAGKLILLPPS--------LDDLKKIAGEK : 764
os_AKT2  : ----------------------------AHWPRVSIYRGHPFVR-NRSS-EAGKLINLPGT--------MEEFRIIIEEK : 805
at_GORK  : --------------------------------KCTVFPFHPQEAKEERSRKHGVVVWVPSN--------LEKLIVTAAKE : 779
at_SKOR  : --------------------------------KCTVYFSHPGDSKEKRRR--GIVLWVPRS--------IEELIRTAKEQ : 793
os_ORK1  : --------------------------------RCSVFPHHPWDGGERRRE--GVVVWIPHT--------IEGLVSSAQEK : 809
os_ORK2  : -------------------------------------------------------------------------------- :   -
                                                                     
                                                                      
                    *       980         *      1000         *         
os_KAT1b : ----------------------------------------------------- :   -
os_KAT1c : QEGFCGGLSVTKLY-NMLNFQLGLELCRIKRNITDLIP--------------- : 747
os_KAT1a : FQGFHPRKVVSRDY-AEID---DVSVIRDGDHLFLLEM--------------- : 710
at_KAT1  : FGGCNFTKITNADN-AEID---DLDVIWDGDHLYFSSN--------------- : 677
at_KAT2  : FG-YSFTKVTNAEN-AEID---DEDVIRDGDHLYILINENS------------ : 565
at_AKT5  : LGFVATKILSREG--AEIDD---IRIIRDGDFLLLLKVS-------------- : 880
at_AKT6  : MGFVPTKVLTREG--AEIDD---ITLIRDGDFLLLSRDP-------------- : 888
at_AKT1  : FGIVATKVMNKDNN-AEIDD---VDVIRDGDHLIFATDS-------------- : 857
os_AKT1a : FDFAPTKVLTVEG--AEVDE---VELIRDGDHLVLVSNEWDAEKMKGKS---- : 943
os_AKT1b : FGVSPTRVVTSGG--ADVDD---ARLVRDGDHLLLVTDKWVPPENRSRNQ--- : 891
os_KAT3b : FGKAVRGILTVEG--AEVED---IEVIRDGDHLLFS----------------- : 591
os_KAT3  : FGKAARGILTVGG--AEVED---IEVIRDGDHLFFSW---------------- : 593
at_KAT3a : LGKRGSTIAMADG--AHVEQ---IDALRENDHLYIF----------------- : 662
at_AKT2  : FGFDGSETMVTNEDGAEIDS---IEVIRDNDKLYFVVNKII------------ : 802
os_AKT2  : LKVDARKTLIMNDEGAEIDS---IDVIRDNDKLFIVTEEHMTAVASMDSVSGS : 855
at_GORK  : LGLS-DGASFVLLSEDQGRIT-DIDMISDGHKLYMISDTTDQT---------- : 820
at_SKOR  : LNVP-EAS--CVLSEDEAKII-DVDLISDGQKLYLAVET-------------- : 828
os_ORK1  : LGLAGSGEGLRLLGEDGARVL-DVDMVHDGQKLYLVVGGGGDDGGTEARQ--- : 858
os_ORK2  : ----------------------------------------------------- :   -
                                      

Sequence Alignment for Additional File 1b

                      *        20         *        40         *        60         *              
os_CNGC1c  : ------------------------------------------------------------------------------ :   -
os_CNGC1b  : ------------------------------------------------------------------------------ :   -
os_CNGC1a  : ------------------------------------------------------------------------------ :   -
at_CNGC1   : ------------------------------------------------------------------------------ :   -
at_CNGC12  : ------------------------------------------------------------------------------ :   -
at_CNGC11  : ------------------------------------------------------------------------------ :   -
at_CNGC3   : ------------------------------------------------------------------------------ :   -
at_CNGC13  : ------------------------------------------------------------------------------ :   -
at_CNGC10  : ------------------------------------------------------------------------------ :   -
at_CNGC6   : ------------------------------------------------------------------MFDTCGPKGVKS :  12
at_CNGC9   : ------------------------------------------------------------------MLD-CGKKAVKS :  11
at_CNGC5   : ------------------------------------------------------------------------------ :   -
at_CNGC8   : ------------------------------------------------------------------------------ :   -
at_CNGC7   : ------------------------------------------------------------------------------ :   -
os_CNGC5a  : ----------------------------------------------------------------------MFDSAHKA :   8
os_CNGC5c  : -------------------------------------------------------------------MSYASGGGGGG :  11
os_CNGC5b  : -------------------------------------------------------------------MFGSCGGGYRT :  11
os_CNGC17c : ------------------------------------------------------------------------------ :   -
os_CNGC17b : ------------------------------------------------------------------------------ :   -
at_CNGC17  : ------------------------------------------------------------------------------ :   -
at_CNGC14  : ------------------------------------------------------------------------------ :   -
os_CNGC18  : ------------------------------------------------------------------------------ :   -
at_CNGC18  : ------------------------------------------------------------------------------ :   -
at_CNGC16  : ------------------------------------------------------------------------------ :   -
os_CNGC14a : ---------------------------------------------------------------------MFGSRVQDE :   9
os_CNGC14b : ------------------------------------------------------------------------------ :   -
os_CNGC15  : ------------------------------------------------------------------------------ :   -
at_CNGC15  : ------------------------------------------------------------------------------ :   -
at_CNGC2   : ---------------------------------------------------MPSHPNFIFR----WIGLFSDKFRRQT :  23
os_CNGC2   : ---------------------------------------------------MPSLSFLRFLSGRSLADVCDGVKRRLG :  27
os_CNGC4a  : ------------------------------------------------------------------------------ :   -
os_CNGC4b  : ------------------------------------------------------------------------------ :   -
at_CNGC4   : ------------------------------------------------------------------------------ :   -
os_CNGC19b : -MSGQERDDVPML---------ELQRFPTR--SVSMCIPVR--DDIYEDS-IISHSGPIFTPA--PTQYTSVAIPSGN :  61
os_CNGC19a : -MSDQERDDIPMLLR-----NVELPTFPPR--STSMCIPVR--DDEYEEDTFVPHTGPLFVQP--PTQTAAAGIPFTN :  66
at_CNGC19  : --------------------MAHTRTFTSRNRSVSLSNPSFSIDGFDNSTVTLGYTGPLRTQRIRPPLVQMSGPIHST :  58
at_CNGC20  : MASHNENDDIPMLPISDPSSRTRARAFTSRSRSVSLSNPTSSIEGFDTSTVVLGYTGPLRTQR-RPPLVQMSGPLTST :  77
                                                                                                 
                                                                                                 
             80         *       100         *       120         *       140         *            
os_CNGC1c  : -MPCLLSHFVVSFEDWRSKQSVMSLR-----------------RHNALSSLKERTAG---------------IFAFLG :  45
os_CNGC1b  : -MMMGREDKYVRFEDWRSEQSVMSPR-----------------RHNALSSLKERTAG---------------VFAFLG :  45
os_CNGC1a  : --MAFREDKYVRFHDWRSEHSVGSEKTVL------------EGRHNVFDSLMDRTVG---------------AFSFLG :  49
at_CNGC1   : --MNFRQEKFVRFQDWKSDKTSSDVEYS--------------GKNEIQTGIFQRTISSISDKFYRSFESSSARIKLFK :  62
at_CNGC12  : --------------------------------------------MGVDGKLKSVRGR------------------LKK :  16
at_CNGC11  : ------------------------------------------------------------------------------ :   -
at_CNGC3   : ------MMNPQRNKFVRFNGNDDEFSTK-------------TTRPSVSSVMKTVRRS------------------FEK :  41
at_CNGC13  : --MAFGRNNRVRFRDWISEGTEYGYGRN-------------KARPSLNTVLKNVRRG------------------LKK :  45
at_CNGC10  : --MILFRFK----DEGKPLSSEYGYGR--------------KARPSLDRVFKNVKWG------------------FKK :  40
at_CNGC6   : QVISGQRENFVRLDSMDSRYSQSSETGLNKCTLNIQGGPKR-FAQGSKASSGSFKKGFR------------KGSEGLW :  77
at_CNGC9   : QVISGRLEKFVRLDSMDSRYSQTSDTGLNRCTLNLQGPTRGGGAQGNNVSSGSFKKGFR------------KGSKGLW :  77
at_CNGC5   : --MAGKRENFVRLPSSSVAFQQNYAS-------NFSGQLHP--IHASNETSRSFKKGIQ------------KGSKGLK :  55
at_CNGC8   : --MSSNATGMKKRSCFGLFNVTSRGG------------------GKTKNTSKSFREGVK------------IGSEGLK :  46
at_CNGC7   : --------MMMQRNCFG-FNLKNRGG------------------EKKK-ASKSFREGVKK-----------IRSEGLI :  39
os_CNGC5a  : QYIDGQREMFKRLDESSPRSSVPSEVG-GRSTLKFSMPSFG---YDSFNPVRSFLSGVR------------KGSGRLK :  70
os_CNGC5c  : ELATKRSAFHIDYGGGVSLRRLAQPEALARG-----------MITQGSAQLRTLGRSLR------------TGAA--- :  63
os_CNGC5b  : QTINGRKGTFVRLEQQEDQERQPAATYTMDGSGGGGRVQHV-MDSYFSSAPKIRTRSVR------------MAAAGVM :  76
os_CNGC17c : --MELRKQRTVRFHEERAKPT-----------------IPTHQKQAGLATS-KLGLGISE------------------ :  40
os_CNGC17b : ------------------------------------------------------------------------------ :   -
at_CNGC17  : --MELRKDKLLMFYSEGKESK-----------------EAKWAVNDPMSKSYKLSLPSAL------------------ :  41
at_CNGC14  : ------MKQEVLPKKSKTRLK-----------------IPRFGRFKVFPENFEIER---------------------- :  33
os_CNGC18  : ------------------------------------------------------------------------------ :   -
at_CNGC18  : ----MNKIRSLRCL-LPETIT-----------------SASTAASNRGSDGSQFSVLWR------------------- :  37
at_CNGC16  : -MSNLHLYTSARFRNFPTTFS-----------------LRHHHNDPNNQRRRSIFSKLR------------------- :  41
os_CNGC14a : VEMQRRTTNRIFPDERQDQFK-----------------LPFQAARADRFGVNRIDAKTTE------------------ :  52
os_CNGC14b : --MR---LNLIENEARHRQNN-----------------LATGS-------VKTMSSKIVL------------------ :  31
os_CNGC15  : --MACNGSRAVRFQNDMELPHWKTSSVPECTSSSRSTKHGKAQHQQQQHHDPRKWRRGGG------------------ :  58
at_CNGC15  : --MGYGNSRSVRFQEDQEVVHGGESGV-----------KLKFKINGTQINNVKMMSKGK------------------- :  46
at_CNGC2   : TGIDENSNLQINGGDSSSSGSDETPVLSS-VECYACTQVGVPAFHSTSCDQAHAPEWRASAG---------------- :  84
os_CNGC2   : LGDDEGRDEEAGLAGGSSRPAAAAAVAGPPGECYACTQPGVPSFHSTTCDQVHSPDWDADAG---------------- :  89
os_CNGC4a  : ------------MASSSAAAASSAHGVGVVQRLWLEEQERKPPP---------------------------------- :  32
os_CNGC4b  : ------------MEGRRVARCRPSA-------VWCCGGGSGX------------------------------------ :  23
at_CNGC4   : ------------MATEQEFTRASRFSRDSSSVGYYSEEDNTEEEDEEEEEMEEIEEEEEEE----------------- :  49
os_CNGC19b : R-DMLDKLPRPKVKSKPHVVTPEEVGI------SNWPYDQHVPKNKHLMMYSEPLGLCDNPDCVDCPRACK---NKRH : 129
os_CNGC19a : TPDMPPRPPQGKQVNKPHAIMPEEIGG------NRWSYSGNVPKNEHLMMS-GPLGQCDDPDCVNCPPACK---NKRH : 134
at_CNGC19  : RRTEPLFSPSPQ-ESPDSSSTVDVPP-----------EDDFVFKNANLLRS-GQLGMCNDPYCTTCPSYYNRQAAQLH : 123
at_CNGC20  : RKHEPLFLPHPSSDSVGVSSQPERYPSFAALEHKNSSEDEFVLKHANLLRS-GQLGMCNDPYCTTCPSYYNRKAAQIP : 154
                                                                                                 
                                                                                                 
              160         *       180         *       200         *       220         *          
os_CNGC1c  : NLV-----HSETLERSVLHEMKLTTG-----------TLHPQ--GPFLQSWNKIFVLSCIFAVSVDPLFFYIPVINEN : 105
os_CNGC1b  : NLV-----HSETLKRLVLHERKLTTR-----------TLHPQ--GPFLQSWNKIFVLSCIFAVSVDPLFFYIPVINDN : 105
os_CNGC1a  : NSS-----HPETLNKPASEEKKSKTR-----------VLDPQ--GPFLQRWNKIFVISCLIAVSVDPLFFYIPVIDGD : 109
at_CNGC1   : RSY-----KSYSFKEAVSKGIGSTHK-----------ILDPQ--GPFLQRWNKIFVLACIIAVSLDPLFFYVPIIDDA : 122
at_CNGC12  : VYG-----KMKTLEN-----------------------------------WRKTVLLACVVALAIDPLFLFIPLIDSQ :  54
at_CNGC11  : ---------MKTLEN-----------------------------------WRKTVLLACVVALAIDPLFLFIPLIDSQ :  34
at_CNGC3   : GSE-----KIRTFKRPLSVHSNKNKENNKKKK--ILRVMNPN--DSYLQSWNKIFLLLSVVALAFDPLFFYIPYVKPE : 110
at_CNGC13  : PLS-----FGSHNKKRDSNSSTTTQKN----------IINPQ--GSFLQNWNKIFLFASVIALAIDPLFFYIPIVDGE : 106
at_CNGC10  : PLS-----FPSHKDPDHKETSSVTRKN----------IINPQ--DSFLQNWNKIFLFACVVALAIDPLFFYIPIVDSA : 101
at_CNGC6   : SIG--RSIGLGVSRAVFPEDLEVSEKK----------IFDPQ--DKFLLLCNKLFVASCILAVSVDPLFLYLPFINDK : 141
at_CNGC9   : SIG--RSIGLGVSRAVFPEDLKVSEKK----------IFDPQ--DKFLLLCNKLFVTSCILAVSVDPLFLYLPFVKDN : 141
at_CNGC5   : SIG--RSLGFGVYRAVFPEDLKVSEKK----------IFDPQ--DKFLLYCNKLFVASCILSVFVDPFFFYLPVINAE : 119
at_CNGC8   : TIG--KSFTSGVTRAVFPEDLRVSEKK----------IFDPQ--DKTLLLWNRMFVISCILAVSVDPLFFYLPIVDNS : 110
at_CNGC7   : TIG--KS----VTRAVFPEDLRITEKK----------IFDPQ--DKTLLVWNRLFVISCILAVSVDPLFFYLPIVDNS :  99
os_CNGC5a  : SLR--QSLTSGAPKTAFAEDLKSFKKT----------IFDPQ--EKFLFQMNWFCFLSCVFAVAVDPLFFFLPIIDGD : 134
os_CNGC5c  : ------------MAVVFQEDLKNTSRK----------IFDPQ--DRLLVRLNRSFVVSCIVSIAVDPVFFYAPQVTAN : 117
os_CNGC5b  : SIGGYRAERLKSIGRVFQEDLTNMSQK----------IFDPQ--DAFLVRMNRLFVMACIVSVAVDPLFFYLPAVTAT : 142
os_CNGC17c : --------KNKIFLAGNEL--------------WYKKIIDPS--SDFILTWNYVLRIACFVALFMDPLYFYVPKIYYG :  94
os_CNGC17b : ------------------------------------------------------------------------------ :   -
at_CNGC17  : --------RPDNLLPGNRLRYTDASKSKSSKVSWYKTILDPG--SEIVLKWNWVFIVSCMVALFIDPLYFFVPAIGGD : 109
at_CNGC14  : -----------------------------------DKILDPG--GDAVLQWNRVFLFWCLVALYVDPLFFFLSSVKRI :  74
os_CNGC18  : ------------------------------------------------------------------------------ :   -
at_CNGC18  : -----------------------------------HQILDPD--SNIVTYWNHVFLITSILALFLDPFYFYVPYVGG- :  77
at_CNGC16  : -----------------------------------DKTLDPG--GDLITRWNHIFLITCLLALFLDPLYFYLPIVQAG :  82
os_CNGC14a : --------KIKVISEGNIP--------------WHRRILDPG--SSMVLMWNRVFLGSCLFALFIDPFFYYLPLVHVL : 106
os_CNGC14b : --------KLKDFTRIWIS--------------REESMLDPG--GNVVLMWNRVFLVSCVASHFIDPLFFFLPIVERR :  85
os_CNGC15  : --------GGGSLKDRVLSRAFSEELESLMSSGANHLFFDPR--GQLIHLWSKIFLAACLASLFVDPLFLYLTGTRQ- : 125
at_CNGC15  : -----------FLKAKVLSRVFSEDLERVKTK-----ILDPR--GQTIRRWNKIFLIACLVSLFVDPLFFFLPVMRN- : 105
at_CNGC2   : --------SSLVPIQEG-SVPNPARTRFRRLKGPFGEVLDPR--SKRVQRWNRALLLARGMALAVDPLFFYALSIGRT : 151
os_CNGC2   : --------SSLVPVQAQPSAAHHAAAAAARWV--FGPVLDPR--SKRVQRWNRWILLARAAALAVDPLFFYALSIGR- : 154
os_CNGC4a  : ---------------------KRGGGKRRWAWAPL----EPRRAGWWAREWDRAYLLACAAGLMVDPLFLYAVSVSG- :  84
os_CNGC4b  : ---------------------RRLGGGAAASWAAA----DPR--ARWVREWNRAYLLACAAGLMVDPLFLYAVSLSG- :  73
at_CNGC4   : --------EEEDPRIGLTCGGRRNGSSNNNKWMMLGRILDPR--SKWVREWNKVFLLVCATGLFVDPLFLYTLSVSD- : 116
os_CNGC19b : FQRSLAPFDNKFHNILYGYGDRWKKKAGHYLS-YIP-IMKPH--DKAVHRWNQFFVISCLLAIFNDPLFFFLLSVDKD : 203
os_CNGC19a : FHRGSSTLDSKFHNFLCEHGGGWKKEIERFLS-RIP-VMNPH--AKVVQQWNQFFVISCLVAIFIDPLFFFLLSVQKD : 208
at_CNGC19  : TSRVSA---SRFRTVLYGDARGWAKRFASSVRRCLPGIMNPH--SKFVQVWTRVLAFSSLVAIFIDPLFFFLLLIQQD : 196
at_CNGC20  : TSRVSALFDSTFHNALYDDAKGWARRFASSVNRYLPGIMNPH--AKEVQTWTKFFALSCLLAIFIDPLFFFLIKVQEQ : 230
                                                     
                                                                                                 
                240         *       260         *       280         *       300         *        
os_CNGC1c  : -----NTCWYLDKKLEITASVLRFFTDIFYILHIIFQFRTGYIASSP-----------TTFDRGVLVEDRYAIAKR-- : 165
os_CNGC1b  : -----NTCWYLDKKLEITASVLRFFTDIFYILHIIFQFRTGYIASSL-----------TTFGRGVLVEDRYAIAKR-- : 165
os_CNGC1a  : -----NICLYLDKKLEIIASVLRFFTDIFYLLHIIFQFRTGFIAPSS-----------RVFGRGVLVEDTFAIAKR-- : 169
at_CNGC1   : -----KKCLGIDKKMEITASVLRSFTDVFYVLHIIFQFRTGFIAPSS-----------RVFGRGVLVEDKREIAKR-- : 182
at_CNGC12  : -----RFCFTFDKTLVAVVCVIRTFIDTFYVIHIIYYLITETIAPRS-----------QASLRGEIVVHSKATLKT-- : 114
at_CNGC11  : -----RFCFTFDKTLVAVVCVIRTFIDTFYVIHIIYYLITETIAPRS-----------QASLRGEIVVHSKATLKT-- :  94
at_CNGC3   : -----RFCLNLDKKLQTIACVFRTFIDAFYVVHMLFQFHTGFITPSS-----------SGFGRGELNEKHKDIALR-- : 170
at_CNGC13  : -----RHCLNLHRNLEIAASVLRTFIDAFYIIHIVFQFRTAYISPSS-----------RVFGRGELVDDPKAIAIK-- : 166
at_CNGC10  : -----RHCLTLDSKLEIAASLLRTLIDAFYIIHIVFQFRTAYIAPSS-----------RVFGRGELVDDAKAIALK-- : 161
at_CNGC6   : -----AKCVGIDRKLAIIVTTIRTVIDSFYLFHMALRFRTAYVAPSS-----------RVFGRGELVIDPAQIAKR-- : 201
at_CNGC9   : -----EKCIGIDRKLAIIATTLRTVIDAFYLFHMALRFRTAFVAPSS-----------RVFGRGELVIDPAQIAKR-- : 201
at_CNGC5   : -----SKCLGIDRKLAITASTLRTFIDVFYLAHMALQLRTAYIAPSS-----------RVFGRGELVIDPAQIAKR-- : 179
at_CNGC8   : -----KNCIGIDSKLAVTTTTLRTIIDVFYLTRMALQFRTAYIAPSS-----------RVFGRGELVIDPAKIAER-- : 170
at_CNGC7   : G----SSCIGIDTKLAVTTTTLRTIVDVFYLTRMALQFRTAYIAPSS-----------RVFGRGELVIDPAKIAER-- : 160
os_CNGC5a  : DK---SSCIGIDKKLAVTSTIIRTILDLVYLIRVFLQFRTAYVAPSS-----------RVFGTGELVIDPMRIAIR-- : 196
os_CNGC5c  : GG---NLCVGISRDLAISASVVRTVVDLFFAARIVLQFRTAYIAPSS-----------RVFGRGELVIDTAQIAAR-- : 179
os_CNGC5b  : DS---NTCIGFDRGLATGATAVRSAIDLFYLARIALQFRTAYIAPSS-----------RVFGRGELVIDPAAIARR-- : 204
os_CNGC17c : TP---NSCIGRDTRLAIIVTVFRSITDLFYVLQIIIKFRTAYINPSSTL---------GVFSRGDLVTDPGNIAKH-- : 158
os_CNGC17b : ------------------------------------------------M---------RVFGRGDLITDPKEIAWQ-- :  19
at_CNGC17  : KN---YPCARTDTSLSILVTFFRTIADLFYLLHIFIKFRTGFIAPNSST---------RVFGRGELVMDPKAIAWR-- : 173
at_CNGC14  : GR---SSCMTTDLKLGIVITFFRTLADLFYVLHIVIKFRTAYVSRTS-----------RVFGRGELVKDPKLIARR-- : 136
os_CNGC18  : ----------MDMQIGVGVTAVRTVADLFYLAHMILKFRTAFVAPSS-----------RVFGRGELVRDPDQIAIR-- :  55
at_CNGC18  : -----PACLSIDISLAATVTFFRTVADIFHLLHIFMKFRTAFVARSS-----------RVFGRGELVMDSREIAMR-- : 137
at_CNGC16  : -----TACMSIDVRFGIFVTCFRNLADLSFLIHILLKFKTAFVSKSS-----------RVFGRGELVMDRREIAIR-- : 142
os_CNGC14a : DESTNRSCIAKDRRLSITITVLRTFADLFYMLNIMVKFHTAYVDPKS-----------RVLGKGELVLDLKKIQRR-- : 171
os_CNGC14b : DR---QLCMTMDHHLAIILTCLRSFLDIFFIAHIAISFSTAHVDPSS-----------KVLGRGELVTDPKKIANR-- : 147
os_CNGC15  : -----NMCIELKYSLAFTLSMIRSLLDLFYAAHIFFRFRTAFIAPSS-----------RVFGRGELVIQPCKIARR-- : 185
at_CNGC15  : -----EACITIGVRLEVVLTLIRSLADAFYIAQILIRFRTAYIAPPS-----------RVFGRGELVIDSRKIAWR-- : 165
at_CNGC2   : ---TGPACLYMDGAFAAVVTVLRTCLDAVHLWHVWLQFRLAYVSRES-----------LVVGCGKLVWDPRAIASHYA : 215
os_CNGC2   : ---AGQPCVYMDAGLAAAVTALRTAADLAHLAHVLLQFRVAYVSRES-----------LVVGCGKLVWDPRAIAAHYA : 218
os_CNGC4a  : ----PLMCVFLDGWFAAAVTVLRCTVDAMHAWNLLMRLRAAVRPPEEDDGADEEVAAERGAGGNGGGPAPAQVARP-- : 156
os_CNGC4b  : ----PLMCVFLDGWLAAAVTALRCMVDAMHAWNIVTQLRVSR------------------AGRERACAAGPDEEQP-- : 127
at_CNGC4   : ----TCMCLLVDGWLALTVTALRSMTDLLHLWNIWIQFKIARRWPYPGGDSD-------GDTNKGGGTRGSTRVAPPY : 183
os_CNGC19b : -----YKCIVFNWNFAIALAVGRSVTDAIYFLHMLLQFRLAYVAPES-----------RVVGTGDLVDEPMKIAMR-- : 263
os_CNGC19a : -----NKCIVLNWHFATALAVVRSVTDAIYFLHMLLQFRLAYVAPES-----------RVVGAGDLVDEPKKIAVR-- : 268
at_CNGC19  : -----NKCIAIDWRATKVLVSLRSITDLIFFINILLQFRLAYVAPES-----------RIVGAGQLVDHPRKIARH-- : 256
at_CNGC20  : -----NKCIMIDWPMTKAFVAVRSVTDVIFTMNILLQFRLAYVARES-----------TVVGAGQLVSHPKKIALH-- : 290
                              
                                                                                                 
                  320         *       340         *       360         *       380         *      
os_CNGC1c  : YLSTYFLIDVFAVLPLP--------------------------------------------------------QVVIL : 187
os_CNGC1b  : YLSTYFLIDVFAVLPLP--------------------------------------------------------QVVIL : 187
os_CNGC1a  : YLSTYFLIDFLAVLPLP--------------------------------------------------------QVLVL : 191
at_CNGC1   : YLSSHFIIDILAVLPLP--------------------------------------------------------QMVIL : 204
at_CNGC12  : RLLFHFIVDIISVLPIP--------------------------------------------------------QVVVL : 136
at_CNGC11  : RLLFHFIVDIISVLPIP--------------------------------------------------------QVVVL : 116
at_CNGC3   : YLGSYFLIDLLSILPIP--------------------------------------------------------QVVVL : 192
at_CNGC13  : YLSSYFIIDLLSILPLP--------------------------------------------------------QLVVL : 188
at_CNGC10  : YLSSYFIIDLLSILPLP--------------------------------------------------------QIVVL : 183
at_CNGC6   : YLQQYFIIDLLSVLPVP--------------------------------------------------------QIIVW : 223
at_CNGC9   : YLQQYFIIDFLSVLPLP--------------------------------------------------------QIVVW : 223
at_CNGC5   : YLQRWFIIDFLSVLPLP--------------------------------------------------------QIVVW : 201
at_CNGC8   : YLTRYFIVDFLAVLPLP--------------------------------------------------------QIAVW : 192
at_CNGC7   : YLTRYFVVDFLAVLPLP--------------------------------------------------------QIAVW : 182
os_CNGC5a  : YLKSYFVMDFFALLPLP--------------------------------------------------------QIVVW : 218
os_CNGC5c  : YFRRFFAADLLSVLPLP--------------------------------------------------------QIVIW : 201
os_CNGC5b  : YVRRFFVVDLLSVLPLP--------------------------------------------------------QIPIW : 226
os_CNGC17c : YLRSSFVVDLVASLPLPQLKRASALLYLKF-------------------------------------------EIIIW : 193
os_CNGC17b : YLRSDFVVDAVAALPLP--------------------------------------------------------QILIW :  41
at_CNGC17  : YIKSDFIIDLIATLPLP--------------------------------------------------------QIVIW : 195
at_CNGC14  : YLRSDFIVDLIACLPLP--------------------------------------------------------QIVSW : 158
os_CNGC18  : YLKNDFIIDLAAMLPIP--------------------------------------------------------QVIIW :  77
at_CNGC18  : YLKTDFLIDVAAMLPLP--------------------------------------------------------QLVIW : 159
at_CNGC16  : YLKSEFVIDLAATLPLP--------------------------------------------------------QIMIW : 164
os_CNGC14a : YLRTDFFIDLLATIPLP--------------------------------------------------------QVTVW : 193
os_CNGC14b : YIRTNFFIDLVAALPVP--------------------------------------------------------QVLVW : 169
os_CNGC15  : YLAGTFWFDLVTALPLP--------------------------------------------------------QFVIW : 207
at_CNGC15  : YLHKSFWIHLVAALPLP--------------------------------------------------------QVLIW : 187
at_CNGC2   : RSLTGFWFDVIVILPVPQA--------------------------------------------------------VFW : 237
os_CNGC2   : RSLKGLWFDLFVILPIPQHNRYSPFTSPLIPLCVAVFYLRPFTVLGRYDRYLPTFFTSTVYFLRGNLGKNNVNKVIFW : 296
os_CNGC4a  : VSRKGLMLDMFVILPVMQV--------------------------------------------------------IVW : 178
os_CNGC4b  : EDNTPFINRLKSVHVKNEV--------------------------------------------------------VVW : 149
at_CNGC4   : VKKNGFFFDLFVILPLPQV--------------------------------------------------------VLW : 205
os_CNGC19b : YLRGFFVLDLFVVLPLP--------------------------------------------------------QVMIL : 285
os_CNGC19a : YLRGYFLLDFFVVLPLP--------------------------------------------------------QVMIL : 290
at_CNGC19  : YFRGKFLLDMFIVFPIP--------------------------------------------------------QIMIL : 278
at_CNGC20  : YLKGKFFLDLFIVMPLP--------------------------------------------------------QILIL : 312
             
                                                                                                 
                    400         *       420         *       440         *       460              
os_CNGC1c  : VVLPNLRSSEVAKAKN-ILMFIVLCQYVPRLIRIRPLYLQITRS--AGVITETPWPGAVLILLIYLLASH-------- : 254
os_CNGC1b  : VVLPNLGGSEVTKAKN-ILMFIVICQYVPRLIRIRPLYLQITRS--AGVITETPWAGAVLNLLIYLLASHEVITVKIY : 262
os_CNGC1a  : VVLPRLQGSSVMTAKN-ILMVIVICQYVPRLIRIIPLYLQITRS--AGIITETAWAGAAFNLLIYMLASH-------- : 258
at_CNGC1   : IIIPHMRGSSSLNTKN-MLKFIVFFQYIPRFIRIYPLYKEVTRT--SGILTETAWAGAAFNLFLYMLASH-------- : 271
at_CNGC12  : TLIP---LSASLVSER-ILKWIILSQYVPRIIRMYPLYKEVTRA--FGTVAESKWAGAALNLFLYMLHSY-------- : 200
at_CNGC11  : TLIP---LSASLVSER-ILKWIILSQYVPRIIRMYPLYKEVTRA--FGTVAESKRVGAALNFFLYMLHSY-------- : 180
at_CNGC3   : AIVPRMRRPASLVAKE-LLKWVIFCQYVPRIARIYPLFKEVTRT--SGLVTETAWAGAALNLFLYMLASH-------- : 259
at_CNGC13  : AVIPNVNKPVSLITKD-YLITVIFTQYIPRILRIYPLYTEVTRT--SGIVTETAWAGAAWNLSLYMLASH-------- : 255
at_CNGC10  : AVIPSVNQPVSLLTKD-YLKFSIIAQYVPRILRMYPLYTEVTRT--SGIVTETAWAGAAWNLSLYMLASH-------- : 250
at_CNGC6   : RFLYTSRGANVLATKQ-ALRYIVLVQYIPRFLRMYPLSSELKRT--AGVFAETAWAGAAYYLLLYMLASH-------- : 290
at_CNGC9   : RFLYISKGASVLATKR-ALRSIILVQYIPRFIRLYPLSSELKRT--AGVFAETAWAGAAYYLLLYMLASH-------- : 290
at_CNGC5   : RFLQSSNGSDVLATKQ-ALLFIVLVQYIPRFLRVLPLTSELKRT--AGVFAETAWAGAAYYLLLYMLASH-------- : 268
at_CNGC8   : KFLHGSKGTDVLPTKQ-ALLHIVITQYIPRFVRFIPLTSELKKT--AGAFAEGAWAGAAYYLLWYMLASH-------- : 259
at_CNGC7   : KFLHGSKGSDVLPTKT-ALLNIVIVQYIPRFVRFIPLTSELKKT--AGAFAEGAWAGAAYYLLWYMLASH-------- : 249
os_CNGC5a  : RYLHTLDGPDVPSTKN-ALVWVVLFQYIPRLLRIFPVTKDLKRT--AGVFIETAWLGAAYYLLWFMLAGH-------- : 285
os_CNGC5c  : KFLHRSKGAAVLSTKD-ALLIIVFLQYIPRVVRIYPLSSELKRT--SGAFAESAYAGAAYYLLWYMLASH-------- : 268
os_CNGC5b  : NFLHRPKGADLLPTKN-ALLFIVLVQYIPRLVRFYPITSELKRT--TGVFAETAFAGAAYYLLLYMLASH-------- : 293
os_CNGC17c : SVIP-SVKYSLSEHDDDILLLIALFQYVLRLYLVFSLNSKIVEV--TGAFSKTAWQGAAYNLLLYMIAS--------- : 259
os_CNGC17b : FVIP-AIKYSTDEHNNNILVLIVLAQYFPRLYLIFPLTYEIVKT--TGVVAKTAWQGAAYNMLLYMIAS--------- : 107
at_CNGC17  : FVIS-TTKSYRFDHNNNAIALIVLLQYIPRFYLIIPLSSQIVKA--TGVVTKTAWAGAAYNLLLYMLASH-------- : 262
at_CNGC14  : FILP-SIRSSHSDHTTNALVLIVLVQYIPRLYLIFPLSAEIIKA--TGVVTTTAWAGAAYNLLQYMLAS--------- : 224
os_CNGC18  : FVIP-AVNNSSANHTNNTLSMIVLIQYIPRVFLIVSLNSKIVKS--SGVVTRTAWAGAAYNLLLYTLAS--------- : 143
at_CNGC18  : LVIP-AATNGTANHANSTLALIVLVQYIPRSFIIFPLNQRIIKT--TGFIAKTAWAGAAYNLLLYILAS--------- : 225
at_CNGC16  : FVIPNAGEFRYAAHQNHTLSLIVLIQYVPRFLVMLPLNRRIIKA--TGVAAKTAWSGAAYNLILYLLVS--------- : 231
os_CNGC14a : IIMP-SIKNSDYNIRNTTFALVIMIQYIFRMYLIVPLSNQIIKA--AGVVAKSAWLGAAYNLLYYMLAS--------- : 259
os_CNGC14b : IAMP-SIS---FKHINAPFFLIILVQSAIRLYIVILLSLSIMEM--VGFIAKNGWEGAIYSLVLYLVAS--------- : 232
os_CNGC15  : IVIP-KLKESATANRKNILRFSIIFQYLPRLFQIFPLSRQIVMA--TGVMTETAWAGAAYNLILYMLAS--------- : 273
at_CNGC15  : IIIP-NLRGSPMTNTKNVLRFIIIFQYVPRMFLIFPLSRQIIKA--TGVVTETAWAGAAYNLMLYMLAS--------- : 253
at_CNGC2   : LVVPKLIREEKVKLIMTILLLIFLFQFLPKIYHCICLMRRMQKV--TGYIFGTIWWGFALNLIAYFIASH-------- : 305
os_CNGC2   : LVIPKLIREEQIKLIMTMLLLLFLLQFLPKVYHSIYIMRKMQKV--TGYIFGTIWWGFGLNLFAYFIASH-------- : 364
os_CNGC4a  : VAAPAMIRAGSTTAVMTVLLVSFLFEYLPKIYHAVRLLRRMQN----TYVFGTIWWGIALNLMAYFVAAH-------- : 244
os_CNGC4b  : VAAPAMIRAGSTTAVMTVMLVAFMLEYLPKIYHSVVFLRRMQNQ--SGHIFGTIWWGIALNLIAYFVAAH-------- : 217
at_CNGC4   : VVIPSLLKRGSVTLVVSVLLVTFLFQYLPKIYHSIRHLRRNATL--SGYIFGTVWWGIALNMIAYFVAAH-------- : 273
os_CNGC19b : LVIPKYVGLSSANYAKNLLRATVLLQYVPRIIRFVPLLGGQS---TNGFIFESAWSTFVINLLMFVLAGH-------- : 352
os_CNGC19a : LVIPKYVGLSTANYAKNLLRITVLLQYVPRIIRFVPLLGGQSDSSANGFIFESAWANFVINLLMFVLAGH-------- : 360
at_CNGC19  : RIIPLHLGTRREESEKQILRATVLFQYIPKLYRLLPLLAGQT---STGFIFESAWANFVINLLTFMLAGH-------- : 345
at_CNGC20  : WIIPAHLGASGANYAKNLLRAAVLFQYIPKLYRLLPFLAGQT---PTGFIFESAWANFVINLLTFMLAGH-------- : 379

                                                                                                 
              *       480         *       500         *       520         *       540            
os_CNGC1c  : -------VLGALWYLLSIERKDACWRDVCRNNS-TGCN-----QAYLYCGD---KENI-----------FLQTACLPI : 305
os_CNGC1b  : KIKVVILVLGALWYLLSIERKDACWRDMCSNNS-TVCN-----QAYLYCGD---KENS-----------ILRTACLPI : 320
os_CNGC1a  : -------VLGALWYLLSIQREDTCWKDACSR-H-DGCD-----SGSLFCGSNAARNNS-----------FLQDFCPTN : 311
at_CNGC1   : -------VFGAFWYLFSIERETVCWKQACERNN-PPCI-----SKLLYCDPETAGGNA-----------FLNESCPIQ : 325
at_CNGC12  : -------VFGAFWYLSSIERKSKCWRAACART--SDCNL---TVTDLLCKRAGSDNIR-----------FLNTSCPLI : 255
at_CNGC11  : -------VCGAFWYLSSIERKSTCWRAACART--SDCNL---TVTDLLCKRAGSDNIR-----------FLNTSCPLI : 235
at_CNGC3   : -------VFGSFWYLISIERKDRCWREACAKI--QNC-----THAYLYCSPTGEDNRL-----------FLNGSCPLI : 312
at_CNGC13  : -------VFGALWYLISVEREDRCWREACEKIP-EVCN-----FRFLYCDGNSSVRND-----------FLTTSCPFI : 309
at_CNGC10  : -------VFGALWYLISVEREDRCWQEACEKT--KGCN-----MKFLYCENDRNVSNN-----------FLTTSCPFL : 303
at_CNGC6   : -------IVGALWYLLALERNNDCWSKACHNN--QNCT-----RNFLFCG--NQNMKG-YAAWDNIKVSYLQLKCPVN : 351
at_CNGC9   : -------IVGAIWYLLALERYNGCWTKVCSNSS-LDCH-----RNFLFCG--NEKMDG-YAAWTTIKDSVLQLNCPVN : 352
at_CNGC5   : -------IVGAFWYLLALERNDACWQEACIDA--GNCS-----TDFLYCG--NQNMDG-YAVWNRAKESVLKSKCRAD : 329
at_CNGC8   : -------ITGAFWYMLSVERNDTCLRSACKVQPDPKVC-----VQILYCG--SKLMSSRETDWIKSVPDLFKNNCSAK : 323
at_CNGC7   : -------ITGAFWYMLSVERNDTCWRFACKVQPDPRLC-----VQILYCG--TKFVSSGETEWIKTVPELLKSNCSAK : 313
os_CNGC5a  : -------NVGTLWYFLTIEREDSCWRSNCHSN--DGCN-----KSYLYCS--DNHTGN-YTSWLSKRTELLS-ACSTN : 345
os_CNGC5c  : -------IVGASWYLLSIERVSDCWKKACN--EFPGCN-----KIYMYCG--NDHQKG-FLEWRTITRQYINETCEPR : 329
os_CNGC5b  : -------MVGAFWYLLSIERLDDCWRENCRVLKFHQC------KKYMYCGGGNLGQSG-FLEWRTMIRQVLVMECAPA : 357
os_CNGC17c : ------HVLGALWYLLSVDRQTACWEKYCSKEA-------GCQNRYLACDIQSDSN--------WKISTAIFNKCDAT : 316
os_CNGC17b : ------HVC--------------------------------------------------------------------- : 110
at_CNGC17  : -MFLLGQVLGAAWYILSVDRYTSCWKSRCNGEAGQV----NCQLYYLDCDSMYDNNQM-----TWANVTKVFKLCDAR : 330
at_CNGC14  : ------HILGSAWYLLSIERQATCWKAECHKESVPL----QCVTDFFDCGTLHRDDRN-----NWQNTTVVFSNCDPS : 287
os_CNGC18  : ------HVLGALWYLLSIERQYTCWMDVCTRENGTNPAIPKCYMSYLDCKTLEDPIRM-----DWHSRSEIDHQCLLP : 210
at_CNGC18  : ------HVLGAMWYLSSIGRQFSCWSNVCKKDNALR--VLDCLPSFLDCKSLEQPERQ-----YWQNVTQVLSHCDAT : 290
at_CNGC16  : ------HVLGSVWYVLSIQRQHECWRRECIKEMNAT-HSPSCSLLFLDCGSLHDPGRQ-----AWMRITRVLSNCDAR : 297
os_CNGC14a : ------HITGAIYYLLSIERQITCWNQQCLNESCSF--------NFISCDNTGSSSYL-----TWGKNTSIFDNCDPN : 318
os_CNGC14b : ------HVVGAIFYLTAVDRQKTCWETQCSIEDR--------------MAHKG----L-----TY------------- : 268
os_CNGC15  : ------HVLGALWYLFSVQRQEACWREACHVEGPS------CQTLFFDCKTVSS-NRT-----MWYELSNITSLCTPS : 333
at_CNGC15  : ------HVLGACWYLLAVERQEACWRHACNIEKQI------CQYRFFECRRLEDPQRN-----SWFEWSNITTICKPA : 314
at_CNGC2   : -------VAGGCWYVLAIQRVASCIRQQCMRTGNCNLS-----LACKEEVCYQFVSPT-STVGYPCLSGNLTSV--VN : 368
os_CNGC2   : -------IAGGCWYVLAIQRVASCLQEECKIKNTCNLTS----LACSKEMCFHLPWSD-KNG----LACNLTSFGQQN : 426
os_CNGC4a  : -------AVGACWYLLGAQRATKCLKEQCAQGGS-GCAP--GALACAAPLYYGGAVGG-VGADRLAWALDASAR---- : 307
os_CNGC4b  : -------AVGACWYLLGVQRATKCLKEQCLLAGLPACASSTAAVACVDPLYYGAAVAS-VGGDRLAWGGNATAR---- : 283
at_CNGC4   : -------AAGACWYLLGVQRSAKCLKEQCE--NTIGCDLR--MLSCKEPVYYGTTVMV-LDRARLAWAQNHQAR---- : 335
os_CNGC19b : -------VVGSCWYLFGLQRVNQCLRDSCAASNISKAL----------CNNCTDCG-------ITGINRTNWLNNSDL : 406
os_CNGC19a : -------VVGSCWYLFGLQRVNQCLRNACSASKIPS------------CDGFIDCGRGINIGKQNQLSRQQWFNDSAS : 419
at_CNGC19  : -------AVGSCWYLSALQRVKKCMLNAWNISADER-------------RNLIDCARGS---YASKSQRDLWRDNASV : 400
at_CNGC20  : -------VVGSCWYLFGLQRVNQCLRNACGNFGREC-------------QDLIDCGNG----NSSVLVRATWKDNASA : 433
                      
                                                                                                 
                *       560         *       580         *       600         *       620          
os_CNGC1c  : N----SNNIDPNFGIYVPALNN-VSQST--DFLAKLFYCVCWGLQNLSSRGQNLKTSTYAWENLFALFVSISGLVLFA : 376
os_CNGC1b  : D----SNDIDPNFGIYVPALNN-VSQST--NFLAKLFYCVWWGLQNLSSLGQNLKTSTYAWENLFAVFVSISGLVLFA : 391
os_CNGC1a  : G----TDNADPTFGIYLPALQN-VSQST--SFFEKLFYCFWWGLQNLSSLGQNLKTSTYTWENLFAVFVSTSGLVLFA : 382
at_CNGC1   : T----PNTTLFDFGIFLDALQSGVVESQ--DFPQKFFYCFWWGLQNLSSLGQNLKTSTYIWEICFAVFISIAGLVLFS : 397
at_CNGC12  : DPAQITNSTDFDFGMYIDALKSGVLEVKPKDFPRKFVYCFWWGLRNISALGQNLETSNSAGEIFFAIIICVSGLLLFA : 333
at_CNGC11  : DPAQITNSTDFDFGMYIDALKSGVLEVKPKDFPRKFVYCFWWGLRNISALGQNLETSNSAGEIFFAIIICVSGLLLFA : 313
at_CNGC3   : DPEEITNSTVFNFGIFADALQSGVVESR--DFPKKFFYCFWWGLRNLSALGQNLKTSAFEGEIIFAIVICISGLVLFA : 388
at_CNGC13  : NPDDITNSTVFNFGIFTDALKSGIVESD--DFWKKFFYCFWWGLRNLSALGQNLNTSKFVGEIIFAVSICISGLVLFA : 385
at_CNGC10  : DPGDITNSTIFNFGIFTDALKSGVVESH--DFWKKFFYCFWWGLRNLSALGQNLQTSKFVGEIIFAISICISGLVLFA : 379
at_CNGC6   : VPE--DEEPPFDFGIYLRALSSGIVSSK--NFVSKYFFCLWWGLQNLSTLGQGLETSTYPGEVIFSITLAIAGLLLFA : 425
at_CNGC9   : TTD--N--PPFDFGIYLRALSSGIVSSK--SFVSKYFFCLWWGLQNLSTLGQGLETSTYPGEVIFSIALAIAGLLLFA : 424
at_CNGC5   : LDD--NN-PPFDFGIYTQALSSGIVSSQ--NFIVKYCYCLWWGLQNLSTLGQGLETSTYPMEIIFSISLAISGLILFA : 402
at_CNGC8   : SDE--SK---FNYGIYSQAVSSGIVSST--TFFSKFCYCLWWGLQNLSTLGQGLQTSTYPGEVLFSIAIAVAGLLLFA : 394
at_CNGC7   : ADD--SK---FNYGIYGQAISSGIVSST--TFFSKFCYCLWWGLQNLSTLGQGLQTSTFPGEVLFSIAIAIAGLLLFA : 384
os_CNGC5a  : ---------SFQFGIFEQALVSGILRPG--NFISKICYCFWWGLQNLSTLGQGLQTSIYPGEVLFSIAICVIGLILFA : 412
os_CNGC5c  : DGV--MP---FNYGIYTPAVRSDVIKSN--DFTSKLLYCLWWGLANLSTLGQGLQTSIYTGEALFSIFLATFGLILMA : 400
os_CNGC5b  : DEA--GTG--FQYGIFTTAIQSGVVSTT--NLVAKVLFCLWWGLQNLSTVGQGLKTTHYKGEALFAIFLAVFGLILMA : 429
os_CNGC17c : NKTI-----DFDFGMFTPLLSNQAPDQG---FLKKFFYCLWWGLQNLSCYGQTLTVSTYIGETLYAIFLAVLGLVLFA : 386
os_CNGC17b : ------------------------------------------------CYGQTITVSTYIGETLYCIFLAVLGLVLFA : 140
at_CNGC17  : NG-------EFKYGIFGNAITKNVVSSQ---FFERYFYCLWWGLQQLSSYGQNLSTTMFMGETTFAVLIAIFGLVLFA : 398
at_CNGC14  : NN-I-----QFTFGIFADALTKNVVSSP---FLEKYLYCLWFGLQNLSSYGQNLSTSTSVLETMFAILVAIFGLVLFA : 356
os_CNGC18  : EAT-------YVYGLFADALNLDVAKVN---FWDKYLYCLWWGFRNLSSYGQNLENSTYRGETIFCILICIMGLVFFS : 278
at_CNGC18  : SSTT-----NFKFGMFAEAFTTQVATTD---FVSKYLYCLWWGLRNLSSYGQNITTSVYLGETLFCITICIFGLILFT : 360
at_CNGC16  : NDDD-Q---HFQFGMFGDAFTNDVTSSP---FFDKYFYCLWWGLRNLSSYGQSLAASTLSSETIFSCFICVAGLVFFS : 368
os_CNGC14a : RNSS-ANPPPFNYGMFSTALSKGAVSAP---FLEKYFFCLWWGLLQLSSSGNPLQTSAYIAENTFAIAIGALSLVLFA : 392
os_CNGC14b : --------------------------------------------------GNPLVTSSFIGENLFAIGLTLLSIGLFA : 296
os_CNGC15  : NG-------FYQFGIYGEALDNGLTSSS---FTQKYFYCFWWGLKNLSCLGQNLSTSLFIGEITFATVIGVLGLVLFA : 401
at_CNGC15  : SK-------FYEFGIFGDAVTSTVTSSK---FINKYFYCLWWGLKNLSSLGQNLATSTYAGEILFAIIIATLGLVLFA : 382
at_CNGC2   : KPMCLDSNGPFRYGIYRWALPVISSNSL----AVKILYPIFWGLMTLSTFANDLEPTSNWLEVIFSIVMVLSGLLLFT : 442
os_CNGC2   : IPDCLSGNGPFAYGIYKGALPVISSNSL----AVKILYPIFWGLMTLSTFGNDLEPTSNWLEVIFSIINVLSGLMLFT : 500
os_CNGC4a  : -GTCLDSGDNYQYGAYKWTVMLVANPSR----LEKILLPIFWGLMTLSTFGN-LASTTEWLEIVFNIITITGGLILVT : 379
os_CNGC4b  : -NVCLSSGDNYQYGAYKWTVMLVSNPSR----LEKMLLPIFWGLMTLSTFGN-LESTTEWVEIVFNIMTITGGLILVT : 355
at_CNGC4   : -SVCLDINTNYTYGAYQWTIQLVSSESR----LEKILFPIFWGLMTLSTFGN-LESTTEWSEVVFNIIVLTSGLLLVT : 407
os_CNGC19b : TGCFDTKSGNFPYGIYQQAVLLTTEP-----GLKRYIYSLFWGFQQISTLAGNLIPSYFVWEVIFTMAIIGLGLLLFA : 479
os_CNGC19a : TACFDTGDNGFHYGIYEQAVLLTTEDN----AVKRYIYSLFWGFQQISTLAGNLVPSYFAWEVLFTMAIIGLGLLLFA : 493
at_CNGC19  : NACFQE--NGYTYGIYLKAVNLTNESS----FFTRFSYSLYWGFQQISTLAGNLSPSYSVGEVFFTMGIIGLGLLLFA : 472
at_CNGC20  : NACFQE--DGFPYGIYLKAVNLTNHSN----LFTRYSYSLFWGFQQISTLAGNQVPSYFLGEVFFTMGIIGLGLLLFA : 505
                          
                                                                                                 
                  *       640         *       660         *       680         *       700        
os_CNGC1c  : LLIANVQ-----------------------------TYLKSAHLREEEMRVKSRDTDQWMSYRLLPENLKERIRRHEK : 425
os_CNGC1b  : LLIGNVQGYETKDMEPCARFRVFIPIPFGDKTSIILTYLQSAHLREEEMRVKSRDTDQWMSYRLLPENLKERIRRHEK : 469
os_CNGC1a  : LLIGNVQ-----------------------------TYLQSASVRIEEMRVKRRDTEQWMAHRLLPDNLKERILRHEQ : 431
at_CNGC1   : FLIGNMQ-----------------------------TYLQSTTTRLEEMRVKRRDAEQWMSHRLLPENLRKRIRRYEQ : 446
at_CNGC12  : VLIGNVQ-----------------------------KYLQSSTTRVDEMEEKRRDTEKWMSYRVIPEYLKERIRRFED : 382
at_CNGC11  : VLIGNVQ-----------------------------KYLQSSTTRVDEMEEKKRDTEKWMSYREIPEYLKERIRRFED : 362
at_CNGC3   : LLIGNMQ-----------------------------KYLQSTTVRVEEMRVKRRDAEQWMSHRMLPDDLRKRIRKYEQ : 437
at_CNGC13  : LLIGNMQ-----------------------------KYLESTTVREEEMRVRKRDAEQWMSHRMLPDDLRKRIRRYEQ : 434
at_CNGC10  : LLIGNMQ-----------------------------KYLESTTVREEEMRVRKRDAEQWMSHRMLPEDLRKRIRRYEQ : 428
at_CNGC6   : LLIGNMQ-----------------------------TYLQSLTIRLEEMRVKRRDSEQWMHHRMLPPELRERVRRYDQ : 474
at_CNGC9   : LLIGNMQ-----------------------------TYLQSLTIRLEEMRVKRRDSEQWMHHRMLPPELRERVRRYDQ : 473
at_CNGC5   : LLIGNMQ-----------------------------TYLQSLTIRLEEMRVKRRDSEQWMHHRMLPQDLRERVRRYDQ : 451
at_CNGC8   : LLIGNMQ-----------------------------TYLQSLTVRLEEMRIKRRDSEQWMHHRSLPQNLRERVRRYDQ : 443
at_CNGC7   : LLIGNMQ-----------------------------TYLQSLTVRLEEMRIKRRDSEQWMHHRSLPQNLRERVRRYDQ : 433
os_CNGC5a  : LLIGNMQ-----------------------------TYLQSVAIRLEEMRVKKRDAEQWMHHRSLPPQIRERVRRYER : 461
os_CNGC5c  : MLIGNIQ-----------------------------TYLQSMTVRLEEMRVKRRDSEQWMHHRLLPQELRERVRRYDA : 449
os_CNGC5b  : LLIGNMQ-----------------------------TYLQSMTLRLEEMRLRRRDSEQWMRHRVLPVDLQERVWRHDQ : 478
os_CNGC17c : HLIGNVQT-----------------------------YLQSITARVEEWRIKQRDTEEWMRHRQLPQKLRERVRRFVH : 435
os_CNGC17b : HLIGNVQT-----------------------------YLQSITVRVEEWRLKQRDTEEWMRHRQLPHELRERVRRFIQ : 189
at_CNGC17  : HLIGNMQT-----------------------------YLQSLTVRLEEWRLKKRDTEEWMRHRQLPEELRNRVRRYEQ : 447
at_CNGC14  : LLIGNMQT-----------------------------YLQSITVRLEEWRLKRRDTEEWMGHRLLPQNLRERVRRFVQ : 405
os_CNGC18  : HLIGNMQT-----------------------------YLQSMTVRLEEWRVKRRDIEEWMRHRQLPLELQERVRRFFQ : 327
at_CNGC18  : LLIGNMQS-----------------------------SLQSMSVRVEEWRVKRRDTEEWMRHRQLPPELQERVRRFVQ : 409
at_CNGC16  : HLIGNVQN-----------------------------YLQSTTARLDEWRVRRRDTEEWMRHRQLPDELQERVRRFVQ : 417
os_CNGC14a : QLIGNMQT-----------------------------YLQSISKRLEEWRLRQRDMEEWMRHHQLPDELQDRVRRFVQ : 441
os_CNGC14b : QLIGNMQ----------------------------------------------------------------------- : 303
os_CNGC15  : LLIGNMQ---------------------------------ATMVRLEEWRTKRTDMERWMNHRQIPQPLKQCVRRYHQ : 446
at_CNGC15  : LLIGNMQT-----------------------------YLQSTTMRLEEWRIRRTDTEQWMHHRQLPPELRQAVRKYDQ : 431
at_CNGC2   : LLIGNIQV-----------------------------FLHAVMAKKRKMQIRCRDMEWWMKRRQLPSRLRQRVRRFER : 491
os_CNGC2   : LLIGNIQV-----------------------------FLHAVLARKRKMQLRFRDMEWWMRRRQLPSRLRQRVRKYER : 549
os_CNGC4a  : MLIGNIKV-----------------------------FLNAATSKKQAMQTRLRGVEWWMKRKKLPQSFRHRVRQHER : 428
os_CNGC4b  : MLIGNIKV-----------------------------FLNATTSKKQAMQTRLRGLEWWMEHKGVPHGFRQRVRQFER : 404
at_CNGC4   : MLIGNIKV-----------------------------FLHATTSKKQAMHLKMRNIEWWMKKRHLPIGFRQRVRNYER : 456
os_CNGC19b : LLIGSMQN-----------------------------FLQALGKRRLEMQLRRRDVEQWMSHRRLPEDLRRRVRSAER : 528
os_CNGC19a : LLIGNMQN-----------------------------FLQALGRRRLEMQLRRRDVEQWMSHRRLPEDLRRRVRRAER : 542
at_CNGC19  : RLIGNMHN-----------------------------FLQSLDRRRMEMMLRKRDVEQWMSHRRLPEDIRKRVREVER : 521
at_CNGC20  : LLIGNMQN-----------------------------FLQALGKRNLEMTLRRRDVEQWMSHRRLPDGIRRRVREAER : 554
              
                                                                                                 
                    *       720         *       740         *       760         *       780      
os_CNGC1c  : YRWHQTSGVDEELLLMNLPKDLRRAIKRHLCLSLLMR------------------------------------SNLLY : 467
os_CNGC1b  : YRWHQTSGVDEELLLMNLPKDLRRAIKRHLCLSLLMR------------------------------------VPMFE : 511
os_CNGC1a  : YRWQETRGVDEEGLLSNLPKNLRREIKRHLCLSLLMRMLVLDFLVLCQTFPLLGHS-----------------VPMFE : 492
at_CNGC1   : YKWQETRGVDEENLLSNLPKDLRRDIKRHLCLALLMR------------------------------------VPMFE : 488
at_CNGC12  : YKWRETKGTEEEALLRSLPKDLRLETKRYLYLDMLKR------------------------------------VPWLN : 424
at_CNGC11  : YKWRRTKGTEEEALLRSLPKDLRLETKRYLFLKLLKK------------------------------------VPLLQ : 404
at_CNGC3   : YKWQETKGVEEEALLSSLPKDLRKDIKRHLCLKLLKK------------------------------------VPWFQ : 479
at_CNGC13  : YKWQETRGVEEENLLRNLPKDLRRDIKRHFCLDLLKK------------------------------------VPLFE : 476
at_CNGC10  : YRWQETRGVEEETLLRNLPKDLRRDIKRHLCLDLLKK------------------------------------VPLFE : 470
at_CNGC6   : YKWLETRGVDEENLVQNLPKDLRRDIKRHLCLALVRR------------------------------------VPLFE : 516
at_CNGC9   : YKWLETRGVDEENLVQNLPKDLRRDIKRHLCLALVRR------------------------------------VPLFE : 515
at_CNGC5   : YKWLETRGVDEEYLVQNLPKDLRRDIKRHLCLALVRR------------------------------------VPLFK : 493
at_CNGC8   : YKWLETRGVDEENIVQSLPKDLRRDIKRHLCLNLVRR------------------------------------VPLFA : 485
at_CNGC7   : YKWLETRGVDEENIVQSLPKDLRRDIKRHLCLNLVRR------------------------------------VPLFA : 475
os_CNGC5a  : YRWLETRGVDEENLVQTLPKDLRRDIKRHLCLGLVKR------------------------------------VPLFE : 503
os_CNGC5c  : YKWVNTRGVDEEVLVANLPKDLRRDIKRHLCLGLVRR------------------------------------VPLFA : 491
os_CNGC5b  : YRWLETRGVDEDSLVRSLPKDLRRDVKRHLCLRLVRR------------------------------------VPLFA : 520
os_CNGC17c : YKWLATRGVDEESILKALPADLRRDIKRHLCLDLVCRVISLPFITTFEWTKEVDTRKINRRGNEKNDRIGTCTFSFFP : 513
os_CNGC17b : YKWLATRGVNEESILQALPADLRRDIKRHLCLGLVRRVP------------------------------------FF- : 230
at_CNGC17  : YKWLATRGVDEEVLLQSLPTDLRRDIQRHLCLDLVRRVP------------------------------------FF- : 488
at_CNGC14  : YKWLATRGVDEETILHSLPADLRRDIQRHLCLDLVRRVP---------------------------------LF---- : 446
os_CNGC18  : YKWLATRGVDEESILQSLPLDLRREIQRHLCLALVRRVP------------------------------------FF- : 368
at_CNGC18  : YKWLATRGVDEESILHSLPTDLRREIQRHLCLSLVRRVP------------------------------------FF- : 450
at_CNGC16  : YKWLTTRGVDEEAILRALPLDLRRQIQRHLCLALVRRVP------------------------------------FF- : 458
os_CNGC14a : VKWLATRGVEEESILQALPADIRRDVQRHLCLDLVRRVP------------------------------------FF- : 482
os_CNGC14b : -------GVEEDSILRQLPADLHRDIKRYLCLDLVERVP------------------------------------FF- : 337
os_CNGC15  : YKWLATRGVDEEALLEDLPMDIRRDIKRHLCLDLVRRVP------------------------------------LF- : 487
at_CNGC15  : YKWLATRGVDEEALLISLPLDLRRDIKRHLCFDLVRRVP------------------------------------LF- : 472
at_CNGC2   : QRWNALGGEDELELIHDLPPGLRRDIKRYLCFDLINKVP------------------------------------LFR : 533
os_CNGC2   : ERWAAITGDEEMEMIKDLPEGLRRDIKRYLCLELVKQVP------------------------------------LFH : 591
os_CNGC4a  : QRWAATRGVDECRIVRDLPEGLRRDIKYHLCLDLVRQVP------------------------------------LFQ : 470
os_CNGC4b  : QRWAATRGVDECQIVRDLPEGLRRDIKYHLCLDLVRQVP------------------------------------LFH : 446
at_CNGC4   : QRWAAMRGVDECEMVQNLPEGLRRDIKYHLCLDLVRQVP------------------------------------LFQ : 498
os_CNGC19b : FSWVATRGVNEEELLSNLPEDIQRGIRRHFFGFLKKVR-------------------------------------LFN : 569
os_CNGC19a : FTWAATQGVNEEELLSNLPEDIQRDIRRHFFRFLNKVR-------------------------------------LFT : 583
at_CNGC19  : YTWAATRGVNEELLFENMPDDLQRDIRRHLFKFLKKVR-------------------------------------IFS : 562
at_CNGC20  : FNWAATRGVNEELLFENMPDDLQRDIRRHLFKFLKKVR-------------------------------------IFS : 595
                      
                                                                                                 
                      *       800         *       820         *       840         *       8      
os_CNGC1c  : ------------------------------------------------------------------------------ :   -
os_CNGC1b  : -------------------------NMDD-QLLNALCDRLKPVLYTEGSCIIREEDPVNEMLFIMRGNLMSMTTNGGR : 563
os_CNGC1a  : -------------------------NMDE-KLLDAMCDRLKPMLYTEGSCIIREGDPVNEMLFIMRGNLESMTTNGGQ : 544
at_CNGC1   : -------------------------KMDE-QLLDALCDRLQPVLYTEESYIVREGDPVDEMLFIMRGKLLTITTNGGR : 540
at_CNGC12  : -------------------------IMDDGWLLEAVCDRVKSVFYLANSFIVREGHPVEEMLIVTRGKLKSTTGSHEM : 477
at_CNGC11  : -------------------------AMDD-QLLDALCARLKTVHYTEKSYIVREGEPVEDMLFIMRGNLISTTTYGGR : 456
at_CNGC3   : -------------------------AMDD-RLLDALCARLKTVLYTEKSYIVREGEPVEDMLFIMRGNLISTTTYGGR : 531
at_CNGC13  : -------------------------IMDE-QLLDAVCDKLKPVLYTENSYAIREGDPVEEMLFVMRGKLMSATTNGGR : 528
at_CNGC10  : -------------------------IMDE-QLLDAVCDRLRPVLYTENSYVIREGDPVGEMLFVMRGRLVSATTNGGR : 522
at_CNGC6   : -------------------------NMDE-RLLDAICERLKPCLFTEKSYLVREGDPVNEMLFIIRGRLESVTTDGGR : 568
at_CNGC9   : -------------------------NMDE-RLLDAICERLKPCLYTESSYLVREGDPVNEMLFIIRGRLESVTTDGGR : 567
at_CNGC5   : -------------------------SMDD-KLLDAICMRLKPCLFTESTYLVREGDPVDEMLFIIRGRLESVTTDGGR : 545
at_CNGC8   : -------------------------NMDE-RLLDAICERLKPSLYTESTYIVREGDPVNEMLFIIRGRLESVTTDGGR : 537
at_CNGC7   : -------------------------NMDE-RLLDAICERLKPSLFTESTYIVREGDPVNEMMFIIRGRLESVTTDGGR : 527
os_CNGC5a  : -------------------------NMDE-RLLDAICERLRPTLYTENEYILREGDPVDEMHFILHGCLESETTDGGR : 555
os_CNGC5c  : -------------------------NMDE-RLLDAICERLRPALYTERTFIIREGDPVDQMLFIIRGCLESITTDGGR : 543
os_CNGC5b  : -------------------------NMDE-RLLDAICERLKPSLCTEATYILREGDPVDEMLFIIRGRLESSTTDGGR : 572
os_CNGC17c : FLASYTLDTGSFLVTGQLQLPVNVSLMDG-QLLDAICERLVSSLSTVGTYIVREGDPVTEMLFIIRGKLESSTTDGGR : 590
os_CNGC17b : ------------------------SQMDN-QLLDAICERLVSSLCTQGTYIVREGDPVTEMLFIIRGKLESSTTNGGR : 283
at_CNGC17  : ------------------------SQMDD-QLLDAICERLVSSLCTEGTYLVREGDLISEMLFIIRGRLESSTTNGGR : 541
at_CNGC14  : ------------------------AQMDD-QLLDAICERLASSLSTQGNYIVREGDPVTEMLFIIRGKLESSTTNGGR : 499
os_CNGC18  : ------------------------SQMDE-QLLDAICERLVSSLSTKDAYIVREGDPVSEMLFVIRGELESSTTDGGR : 421
at_CNGC18  : ------------------------SQMDD-QLLDAICGCLVSSLSTAGTYIFREGDPVNEMLFVIRGQIESSTTNGGR : 503
at_CNGC16  : ------------------------AQMDD-QLLDAICERLVPSLNTKDTYVIREGDPVNEMLFIIRGQMESSTTDGGR : 511
os_CNGC14a : ------------------------SEMDY-QLLDAICERLVSFLCPERTYISREGDPVNEMLFVIRGKLESSTTNGGR : 535
os_CNGC14b : ------------------------SAMDH-QLLDAICERMTYFLRTEGTYITREGDPVKVMLFIIRGKLESSTTDGGR : 390
os_CNGC15  : ------------------------DEMDE-RMLEAICERLRPALYTRGTRLVRELDPVDSMLFIIRGYLDSYTTQGGR : 540
at_CNGC15  : ------------------------DQMDE-RMLDAICERLKPALCTEGTFLVREGDPVNEMLFIIRGHLDSYTTNGGR : 525
at_CNGC2   : -------------------------GMDD-LILDNICDRAKPRVFSKDEKIIREGDPVQRMIFIMRGRVK--RIQSLS : 583
os_CNGC2   : -------------------------GMDD-LILDNICDRLRPLVFSSGEKVIREGDPVQRMVFVLQGKLR--STQPLA : 641
os_CNGC4a  : -------------------------HMDD-LVLENICDRVKSLVFPKGEIIVREGDPVQRMLFIVRGHLQ--SSQVLR : 520
os_CNGC4b  : -------------------------HMDD-LVLENICDRVKSLISPKGEIIVREGDPVQRMLFIVRGHLQ--CSQVMR : 496
at_CNGC4   : -------------------------HMDD-LVLENICDRVKSLIFTKGETIQKEGDAVQRMLFVVRGHLQ--SSQLLR : 548
os_CNGC19b : --------------------------LMDNATWDAICDKLRQNLYITGSDILYQGGPVEKMVFIVRGRLESISADG-- : 619
os_CNGC19a : --------------------------LMDWPILDAICDKLRQNLYISGSDILYQGGPVEKMVFIVRGKLESISADG-- : 633
at_CNGC19  : --------------------------LMDESVLDSIRERLKQRTYIRSSTVLHHRGLVEKMVFIVRGEMESIGEDG-- : 612
at_CNGC20  : --------------------------LMDEPILDAIRERLKQRTYIGSSTVLHRGGLVEKMVFIVRGEMESIGEDG-- : 645
                                        
                                                                                                 
             60         *       880         *       900         *       920         *            
os_CNGC1c  : ------------------------------------------------------------------------------ :   -
os_CNGC1b  : TGFFNSDVLKGGDFCGEELLTWALDPTSVSSLP----------SSTRTVKTMSEVEAFALRAEDLKFVATQFRRLHS- : 630
os_CNGC1a  : TGFFNSNIIKGGDFCGEELLTWALDPTSASNLP----------SSTRTVKTLSEVEAFALRADDLKFVATQFRRLHS- : 611
at_CNGC1   : TGFLNSEYLGAGDFCGEELLTWALDPHSSSNLP----------ISTRTVRALMEVEAFALKADDLKFVASQFRRLHS- : 607
at_CNGC12  : GVRNNCCDLQDGDICG-ELLFN------GSRLP----------TSTRTVMTLTEVEGFILLPDDIKFIASHLNVFQR- : 537
at_CNGC11  : TGFFNSVDLIAGDSCG-DLLTWALYSL-SSQFP----------ISSRTVQALTEVEGFVISADDLKFVATQYRRLHS- : 521
at_CNGC3   : TGFFNSVDLVAGDFCG-DLLTWALDPL-SSQFP----------ISSRTVQALTEVEGFLLSADDLKFVATQYRRLHS- : 596
at_CNGC13  : TGFFNAVYLKPSDFCGEDLLTWALDPQSSSHFP----------ISTRTVQALTEVEAFALAADDLKLVASQFRRLHS- : 595
at_CNGC10  : SGFFNAVNLKASDFCGEDLLPWALDPQSSSHFP----------ISTRTVQALTEVEAFALTAEDLKSVASQFRRLHS- : 589
at_CNGC6   : SGFYNRSLLKEGDFCGDELLTWALDPKSGSNLP----------SSTRTVKALTEVEAFALIADELKFVASQFRRLHS- : 635
at_CNGC9   : SGFFNRSLLKEGDFCGEELLTWALDPKSGSNLP----------SSTRTAKALTEVEAFALIADELKFVASQFRRLHS- : 634
at_CNGC5   : SGFFNRSLLKEGEFCGEELLTWALDPKSGVNLP----------SSTRTVKALTEVEAFALTSEELKFVASQFRRLHS- : 612
at_CNGC8   : SGFFNRGLLKEGDFCGEELLTWALDPKAGSNLP----------SSTRTVKALTEVEAFALEAEELKFVASQFRRLHS- : 604
at_CNGC7   : SGFFNRGLLKEGDFCGEELLTWALDPKAGSNLP----------SSTRTVKALTEVEAFALEAEELKFVASQFRRLHS- : 594
os_CNGC5a  : SGFFNKVQLKEGAFCGDELLTWALDPKSAANFP----------ASTRTVKALTEVEAFALCAEELKFVASQFRRLHS- : 622
os_CNGC5c  : SGFFNRSLLEESDFCGEELLTWALDPKAGLSLP----------SSTRTVRALSEVEAFALHSDELKFVAGQFRRMHS- : 610
os_CNGC5b  : MGFFNRGLLKEGDFCGEELLTWALDPKAAANLP----------LSTRTVKAISEVEAFALHADELKFVAGQFRRLHS- : 639
os_CNGC17c : TGFFNSITLKTGDFCGEELLGWALVPKPTVNLP----------SSTRTVKTIVEVEAFALRAEDLKFVASQFRRLHS- : 657
os_CNGC17b : TGFFNSTTLKSGDFCGEELLGWALVPKPTVNLP----------SSTRTVKALIEVEAFALQAEDLKFVANQFRRLHS- : 350
at_CNGC17  : TGFFNSIILRPGDFCGEELLSWALLPKSTLNLP----------SSTRTVRALVEVEAFALRAEDLKFVANQFRRLHS- : 608
at_CNGC14  : TGFFNSITLRPGDFCGEELLAWALLPKSTVNLP----------SSTRTVRALEEVEAFALQAGDLKFVANQFRRLHS- : 566
os_CNGC18  : TNFFSSITLRPGDFCGEELLTWALMPNPSLNFP----------QSTRTVRSVTEVEAFALRAEDLKYVANQFKRLHS- : 488
at_CNGC18  : SGFFNSTTLRPGDFCGEELLTWALMPNSTLNLP----------SSTRSVRALSEVEAFALSAEDLKFVAHQFKRLQS- : 570
at_CNGC16  : SGFFNSITLRPGDFCGEELLTWALVPNINHNLP----------LSTRTVRTLSEVEAFALRAEDLKFVANQFRRLHS- : 578
os_CNGC14a : SNFFNSIILRPGDFAGEELLTWALLPKTNVHFP----------LSTRTVQSLTEVEAFALRAEDLKFVANQFRRLHS- : 602
os_CNGC14b : TGFFNSIILKPGDFCGEELLTWALLPSSRDSYP----------SSTRTVKTIAELEAFSLQADDIKCVASTFRMMHS- : 457
os_CNGC15  : SGFFNSCRIGAGEFCGEELLPWALDPRPAASLP----------LSTRTVRAVSEVEAFALVADDLRFVASQFRRLHS- : 607
at_CNGC15  : TGFFNSCLIGPGDFCGEELLTWALDPRPVVILP----------SSTRTVKAICEVEAFALKAEDLQFVASQFRRLHT- : 592
at_CNGC2   : KGVLATSTLEPGGYLGDELLSWCLRRPFLDRLP----------PSSATFVCLENIEAFSLGSEDLRYITDHFRYKFAN : 651
os_CNGC2   : KGVVATCMLGAGNFLGDELLSWCLRRPSLDRLP----------ASSATFECVETAQAFCLDAPDLRFITEQFRYKFAN : 709
os_CNGC4a  : TGATSCCTLGPGNFSGDELLSWCMRRPFLERLP----------ASSSTLVTMESTEAFGLEAADVKYVTQHFRYTFTN : 588
os_CNGC4b  : NGATSWCTLGPGNFSGDELLSWCMRRPFMERLP----------ASSSTLVTAESTEAFGLEAGDVKYVTQHFRYTFTS : 564
at_CNGC4   : DGVKSCCMLGPGNFSGDELLSWCLRRPFVERLP----------PSSSTLVTLETTEAFGLDAEDVKYVTQHFRYTFVN : 616
os_CNGC19b : ----NKSPLQEGDVCGEELLSWYLEQSSVNRDGGKIKLHGMRLVAIRTVRCLTNVEAFVLRARDLEEVTSQFSRFLRN : 693
os_CNGC19a : ----SKAPLHEGDVCGEELLTWYLEHSSANRDGGRMRFHGMRLVAIRTVMHSVVSVAEYLSS---MICMTMFTNAGAL : 704
at_CNGC19  : ----SVLPLSEGDVCGEELLTWCL--SSINPDGTRIKMPPKGLVSNRNVRCVTNVEAFSLSVADLEDVTSLFSRFLRS : 684
at_CNGC20  : ----SVLPLYEGDVCGEELLTWCLERSSVNPDGTRIRMPSKGLLSSRNVRCVTNVEAFSLSVADLEDVTSLFSRFLRS : 719
                        
                                                                                                 
              940         *       960         *       980         *      1000         *          
os_CNGC1c  : ------------------------------------------------------------------------------ :   -
os_CNGC1b  : KQLQHTFKFYSQHWRTWAACFIQAAWHRYCRKKIEDSLREKEKRLQFAIVN--------------------------- : 681
os_CNGC1a  : KQLQHTFRFYSQQWRTWAACFIQAAWHRYCRKKLEDTLFEKEKRLQAAIVS--------------------------- : 662
at_CNGC1   : KQLRHTFRYYSQQWKTWAACFIQAAWRRYIKKKLEESLKEEENRLQDALAK--------------------------- : 658
at_CNGC12  : QKLQRTFRLYSQQWRSWAAFFIQAAWRKHCKRKLSKTRDNEN------------------------------------ : 579
at_CNGC11  : KQLQHMFRFYSLQWQTWAACFIQAAWKRHCRRKLSKALREE------------------------------------- : 562
at_CNGC3   : KQLRHMFRFYSVQWQTWAACFIQAAWKRHCRRKLSKALREEEGKLHNTLQN---------------D----------- : 648
at_CNGC13  : KQLQHTFRFYSVQWRTWGASFIQAAWRRHCRRKLARSLTEEEDRFRNAITK-----------RERNA----------- : 651
at_CNGC10  : KQLQHTFRFYSVQWRTWSVSFIQAAWRRYCRRKLAKSLRDEEDRLREALASQD---------KEHNA----------- : 647
at_CNGC6   : RQVQHTFRFYSQQWRTWAACFMQAAWRRYIKRKKLEQLRKEEEEEEAAAA---------------SV----------- : 687
at_CNGC9   : RQVQHTFRFYSQQWRTWAAIFIQAAWRRYVKKKKLEQLRKEEEEGE-------------------------------- : 680
at_CNGC5   : RQVQHTFRFYSHQWRTWAACFIQAAWRRYCKRKKMEEAEAEAAAVSSS------------------------------ : 660
at_CNGC8   : RQVQQTFRFYSQQWRTWAACFIQAAWRRHLRRKIAELRRKEEEEEEMDYEDDEYYDDNMGGMVTRSD----------- : 671
at_CNGC7   : RQVQQTFRFYSQQWRTWASCFIQAAWRRYSRRKNAELRRIEEKEEELGYEDEYDDESDKRPMVITRS----------- : 661
os_CNGC5a  : RQVQHTFRFYSQHWRTWAACFIQAAWRRYYKRKMAEQHRKEEEAANRQSS---------------------------- : 672
os_CNGC5c  : KQVQHTFRFYSQQWRTWAATYIQAAWRRHLKRRAAELRRREEEEEE-AAAIR-------------------------- : 661
os_CNGC5b  : KQLQQTFRFYSQQWRTWASCFIQAAWRRHLKRRAAEQRRREEEEEEEAASAS-------------------------- : 691
os_CNGC17c : RKLQHTFRYYSHHWRTWAACFIQAAWRRYKRRRLAKDLSIRESFFSRRS----------FEDDGSP------------ : 713
os_CNGC17b : KRLQHTFRYYSHHWRTWASCFIQAAWRRYKRRKMARDLSMRESFCSMRSDD------SNGEDDSPP------------ : 410
at_CNGC17  : KKLQHTFRFYSHHWRTWAACFIQAAWRRYKRRVMENNLTAIESMENEEGEVG--EELVVVEEEECV------------ : 672
at_CNGC14  : KKLQHTFRYYSHQWRTWAACFVQVAWRRYKRKKLAKSLSLAESFSSYDEEEA--VAVAATEEMSHEG---EAQSGAKA : 639
os_CNGC18  : KRLQHAFRYYSHQWRSWGACFVQGAWRRYKKRKLARELSKQEELY-YMQGQGGDDGDGHDDSDSAPLLGAGVGAGGDH : 565
at_CNGC18  : KKLQHAFRYYSHQWRAWGACFVQSAWRRYKRRKLAKELSLHESSG-YYYP----DETGYNEEDEE----TREYYYGSD : 639
at_CNGC16  : KKLQHAFRYYSHQWRAWGTCFIQAAWRRYMKRKLAMELARQEEEDDYFYD----DDGDYQFEEDMP------ESNNNN : 646
os_CNGC14a : KKLQHTFRFYSHHWRTWAACFIQAAWRQHQ-RRKLAES-LS-RWESYSWWPEEHPPADKPKQEGTS------------ : 665
os_CNGC14b : KHLQHTFRLHSYQWRTWAARFIQSAWRRRQNRQKMAEVGLSNRWKSFFSLVNDFN---DTRCEDIN------------ : 520
os_CNGC15  : ARIRHRFRFYSHQWRTWAACFIQAAWRRNKRRRASMELRMREG------------------GEARP------------ : 655
at_CNGC15  : KQLRHKFRFYSHQWRTWAACFIQAAWRRHRKRKYKTELRAKEEFH-------------YRFEAATA------------ : 645
at_CNGC2   : ERLKRTARYYSSNWRTWAAVNIQMAWRRRRKRTRGENIGGSMSPV--------------------------------- : 696
os_CNGC2   : EKLKRTARYYSSNWRTWAAVNIQLAWRRYKARTTTDLASAAQPPS--------------------------------- : 754
os_CNGC4a  : DRVRRSARYYSHGWRTWAAVAVQLAWRRYKHRKTLASLSFIRPRR--------------------------------- : 633
os_CNGC4b  : DKVRRSARYYSHGWRTWAAVAVQLAWRRYKHRKTLASLSFIRPRR--------------------------------- : 609
at_CNGC4   : EKVKRSARYYSPGWRTWAAVAVQLAWRRYKHRLTLTSLSFIRPRR--------------------------------- : 661
os_CNGC19b : PLVLGTIRYESPYWKNLAANRIQVAWRYRKRRLKRAEMQRLQ------------------------------------ : 735
os_CNGC19a : LLQLHPTGMSHPTGEPLLQRVFKSHGGIGIGD---------------------------------------------- : 736
at_CNGC19  : HRVQGAIRYESPYWRLRAAMQIQVAWRYRKRQLQRLNTAHSNSNR--------------------------------- : 729
at_CNGC20  : HRVQGAIRYDSPYWRLRAARQIQVAWRYRRRRLHRLCTPQSSYSL--------------------------------- : 764
                      
                                                                                        
               1020         *      1040         *      1060         *      1080         
os_CNGC1c  : --------------------------------------------------------------------- :   -
os_CNGC1b  : --DGA-TTLSFRAAIYASRFAGNMMRILRRN-----ATRKARLKESVPARLLQKPAEPNFAAEEQ---- : 738
os_CNGC1a  : --DGS-SSLSLGAALYASRFAGNMMRILRRN-----ATRKARLQERVPARLLQKPAEPNFFAEDQ---- : 719
at_CNGC1   : --EACGSSPSLGATIYASRFAANILRTIRRSG----SVRKPRMPERMPPMLLQKPAEPDFNSDD----- : 716
at_CNGC12  : --IPQGTQLNLASTLYVSRFVSKALQNRRKDT----ADCSS--SPDMSPPVPHKPADLEFAKAEA---- : 636
at_CNGC11  : ---------------------EGKLHNTLQND----DSGGN--KLNLGAAIYA---------------- : 588
at_CNGC3   : --DSGGNKLNLGAAIYASRFASHALRNLRANA----AARNSR-FPHMLTLLPQKPADPEFPMDET---- : 706
at_CNGC13  : --ASSSS---LVATLYASRFASNALRNLRTNN---------------LPLLPPKPSEPDFSLRNP---- : 696
at_CNGC10  : --ATVSSSLSLGGALYASRFASNALHNLRHNI----SNLPP---RYTLPLLPQKPTEPDFTANHTTDP- : 706
at_CNGC6   : --IAGGSPYSIRATFLASKFAANALRSVHKNRT---AKSTLLLSSTKELVKFQKPPEPDFSAEDH---- : 747
at_CNGC9   : -----GSVTSIRATFLASKFAANALRKVHKNR--------IEAKSTIELVKYQKPSEPDFSADDTS--- : 733
at_CNGC5   : ---TAGPSYSIGAAFLATKFAANALRTIHRNR----------NTKIRDLVKLQKPPEPDFTAD------ : 710
at_CNGC8   : --SSVGSSSTLRSTVFASRFAANALKG-HKLR---------VTESSKSLMNLTKPSEPDFEALDTDDLN : 728
at_CNGC7   : --E---SSSRLRSTIFASRFAANALKG-HRLR---------SSESSKTLINLQKPPEPDFDAE------ : 709
os_CNGC5a  : ----SSHHPSLAATIYASRFAANALRGVHRLR----------SRASPTIVRLPKPPEPDFAVDEAD--- : 724
os_CNGC5c  : ------SSTGLKTTMLVSRFAANAMRGVHRQR--------SRRADEVLMMPMPKPSEPDFG-ADY---- : 711
os_CNGC5b  : ------SSCQITTTVLVSRFAKNAMRGAQRQR--------SRR--DANLIVLPKPPEPDFQTMEY---- : 740
os_CNGC17c : -EHS-----------LVLNAV---RKGAHIIK---------------ELPKFRKPSEPDFSAEHDD--- : 749
os_CNGC17b : -KQN-----------LAMKIMSGSRKGPQNMK---------------ELPKLRKPDEPDFSAEPCE--- : 449
at_CNGC17  : -EESPRTKMNLGVMVLASRFAANTRRGVAAQRVK-----------DVELPRFKKPEEPDFSAEHDD--- : 726
at_CNGC14  : RHHTSNVKPHFAATILASRFAKNTRRTAHKLK-------------DVEIPMLPKPDEPDFSVDD----- : 690
os_CNGC18  : RDGAAAGAAHLGATFLASKFAKNTKK----SAAAHHGKARMEDVSSIKFPKLAKPDEPDFSLSSDDVL- : 629
at_CNGC18  : EEGGSMDNTNLGATILASKFAANTRRGTNQKASSSSTGKKDGSSTSLKMPQLFKPDEPDFSIDKEDV-- : 706
at_CNGC16  : GDENSSNNQNLSATILASKFAANTKRG--VLGNQRGSTRIDPDHPTLKMPKMFKPEDPGFF-------- : 705
os_CNGC14a : ---SSTKTIAESAIAQMHKFASASRRFRADDT---------------AIRRLQKPDEPDFSADHFD--- : 713
os_CNGC14b : ---GSSSTVSHRETVTVSKIASIFKKAQKE-----------------------RPEEPDFSEDHHPE-- : 561
os_CNGC15  : --------------GGSVRCRRHSCDG---------------------KALIKKPMEPDFTVEEED--- : 686
at_CNGC15  : --------------RLAVNGGKYTRSGSDSGM----------------MSSIQKPVEPDFSSE------ : 678
at_CNGC2   : ---------------SENSIEGNSERRLLQYA---------------AMFMSIRPHDHLE--------- : 726
os_CNGC2   : ---------------AGGPDDG--DRRLRHYA---------------AMFMSLRPHDHLE--------- : 782
os_CNGC4a  : ---------------PLSRCSSLGEEKLRLYT---------------AILTSPKPNPNQ-DDLV----- : 666
os_CNGC4b  : ---------------PLSRCSSLGEEKLRLYT---------------AILTSPKPNQD--DDF------ : 640
at_CNGC4   : ---------------PLSRCASLGEDKLRLYA---------------AILTSPKPNPDDFDDY------ : 694
os_CNGC19b : --------------------------------------------------------------------- :   -
os_CNGC19a : --------------------------------------------------------------------- :   -
at_CNGC19  : --------------------------------------------------------------------- :   -
at_CNGC20  : --------------------------------------------------------------------- :   -
                                                                                     
