# Supplementary material for: Cyclic nucleotide binding proteins in the Arabidopsis thaliana and Oryza sativa genomes
Source: BMC Bioinformatics. 2005 Jan 11;6:6. doi: 10.1186/1471-2105-6-6 (PMC545951; doi:10.1186/1471-2105-6-6)
Supplement: Additional File 2 — Cyclic nucleotide binding proteins in Arabidopsis thaliana and Oryza sativa. Proteins containing CNB domains including species, name, aliases, accession number, other domains present (AR indicates ankyrin repeat, CaM indicates calmodulin binding domain and TE indicates type II acyl CoA thioesterase domain), sequence length, residues encompassing the CNB domain and E-value for the CNB domain as determined by SMART [62]. All accession numbers are from NCBI except osCNGC5b, osCNGC5c and osCNGC18 which were obtained from the MIPS Oryza sativa database . [file 1471-2105-6-6-S2.doc]

| Sp. | Protein | Aliases | NCBI  Accession | Other Domains | Length | CNB Domain | E-value |
| --- | --- | --- | --- | --- | --- | --- | --- |
| At | GORK |  | CAC17380 | AR | 820 | 386-504 | 1.0E-18 |
| At | SKOR |  | CAA11280 | AR | 828 | 403-521 | 1.1E-24 |
| At | AKT1 |  | AAB95299 | AR | 857 | 372-489 | 1.9E-27 |
| At | AKT2 |  | AAA97865 | AR | 802 | 394-511 | 1.2E-28 |
| At | AKT5 |  | CAB79967 | AR | 880 | 396-513 | 2.4E-22 |
| At | AKT6 | SPIK | AAD31377 | AR | 888 | 398-515 | 3.4E-24 |
| At | KAT1 |  | AAA32824 |  | 677 | 377-494 | 1.3E-23 |
| At | KAT2 |  | AAA67070 |  | 565 | 245-362 | 4.8E-22 |
| At | KAT3 | KC1, AKT4 | CAB05669 |  | 622 | 406-523 | 1.3E-24 |
| At | CNGC1 |  | AAK43954 | CaM | 716 | 486-617 | 8.0E-18 |
| At | CNGC2 | DND1 | AAC78613 | CaM | 726 | 531-660 | 1.7E-4 |
| At | CNGC3 |  | CAB40128 | CaM | 706 | 477-606 | 8.2E-8 |
| At | CNGC4 |  | AAL15321 | CaM | 694 | 496-625 | 5.1E-3 |
| At | CNGC5 |  | CAB79774 | CaM | 710 | 491-622 | 6.8E-13 |
| At | CNGC6 |  | AAK64058 | CaM | 747 | 514-645 | 6.2E-13 |
| At | CNGC7 |  | NP_173051 | CaM | 709 | 473-604 | 3.6E-17 |
| At | CNGC8 |  | NP_173408 | CaM | 728 | 483-614 | 1.8E-15 |
| At | CNGC9 |  | CAB79774 | CaM | 733 | 513-644 | 6.9E-15 |
| At | CNGC10 | ACBK1 | AAF73128 | CaM | 706 | 468-596 | 2.1E-12 |
| At | CNGC11 |  | AAD20357 | CaM | 588 | 402-531 | 3.6E-12 |
| At | CNGC12 |  | AAD23055 | CaM | 636 | 428-547 | 2.2E-7 |
| At | CNGC13 |  | AAL27505 | CaM | 696 | 474-605 | 8.7E-12 |
| At | CNGC14 |  | AAD23866 | CaM | 690 | 445-576 | 4.9E-13 |
| At | CNGC15 |  | AAD29827 | CaM | 678 | 471-602 | 3.8E-12 |
| At | CNGC16 |  | NP_190384 | CaM | 705 | 457-588 | 2.9E-12 |
| At | CNGC17 |  | CAB81029 | CaM | 726 | 487-618 | 8.8E-12 |
| At | CNGC18 |  | NP_196991 | CaM | 706 | 449-580 | 1.5E-15 |
| At | CNGC19 | CNBT2 | AAF73129 | CaM | 729 | 560-693 | 5.5E-3 |
| At | CNGC20 | CNBT1 | AAF73128 | CaM | 764 | 593-728 | 2.7E-2 |
| At | CNTE1 | ACH2 | AY874170 | TE | 425 | 15-131 | 4.7E-8 |
| At | CNTE2 | ACH1 | NP_563632 | TE | 471 | 59-177 | 1.2E-8 |
| Os | ORK1 |  | BAD45977 | AR | 858 | 425-543 | 4.8E-27 |
| Os | ORK2 |  | CAD40969 | AR | 722 | 386-504 | 3.6E-22 |
| Os | AKT1a |  | BAC00610 | AR | 943 | 431-544 | 1.0E-11 |
| Os | AKT1b |  | BAC24865 | AR | 891 | 388-494 | 3.6E-20 |
| Os | AKT2 |  | AAS90669 | AR | 855 | 391-509 | 1.0E-27 |
| Os | KAT1a | ZmK2.1 | BAD27742 |  | 710 | 366-483 | 4.4E-25 |
| Os | KAT1b |  | BAB90143 |  | 502 | 381-498 | 4.2E-26 |
| Os | KAT1c |  | BAA96192 |  | 747 | 323-440 | 1.5E-18 |
| Os | KAT3a |  | BAD46168 |  | 591 | 349-467 | 4.8E-25 |
| Os | KAT3b |  | BAB68056 |  | 593 | 350-468 | 6.6E-19 |
| Os | CNGC1a |  | BAD29689 | CaM | 719 | 490-621 | 2.3E-17 |
| Os | CNGC1b |  | BAD54193 | CaM | 738 | 509-640 | 3.0E-14 |
| Os | CNGC2 |  | AAK16188 | CaM | 782 | 589-715 | 2.2E-8 |
| Os | CNGC4a |  | BAD53284 | CaM | 666 | 468-597 | 1.3E-5 |
| Os | CNGC4b |  | AAU90233 | CaM | 640 | 444-573 | 9.4E-5 |
| Os | CNGC5a |  | CAD41906 | CaM | 724 | 501-632 | 1.8E-13 |
| Os | CNGC5b |  | 9640.m02720 | CaM | 740 | 518-649 | 2.1E-14 |
| Os | CNGC5c |  | 9631.m04291 | CaM | 711 | 489-620 | 2.5E-16 |
| Os | CNGC14a |  | BAD45941 | CaM | 713 | 481-609 | 6.0E-9 |
| Os | CNGC14b |  | BAC98536 | CaM | 561 | 336-467 | 5.3E-10 |
| Os | CNGC15 |  | BAD23159 | CaM | 686 | 486-617 | 9.5E-8 |
| Os | CNGC17a |  | BAD36523 | CaM | 749 | 536-667 | 2.9E-6 |
| Os | CNGC17b |  | BAD19100 | CaM | 449 | 229-360 | 4.9E-15 |
| Os | CNGC18 |  | 9640.m00579 | CaM | 629 | 367-498 | 7.6E-11 |
| Os | CNGC19a |  | BAD16877 | CaM | 736 | 581-679 | 4.5E-2 |
| Os | CNGC19b |  | BAD35887 | CaM | 735 | 567-702 | 1.8E-4 |
| Os | CNTE |  | CAE03486 | TE | 428 | 15-135 | 1.3E-4 |
